# CircRNA-DOPEY2 enhances the chemosensitivity of esophageal cancer cells by inhibiting CPEB4-mediated Mcl-1 translation

**DOI:** 10.1186/s13046-021-02149-5

**Published:** 2021-11-15

**Authors:** Zhenchuan Liu, Shaorui Gu, Kaiqin Wu, Lei Li, Chenglai Dong, Wenli Wang, Yongxin Zhou

**Affiliations:** Department of Thoracic Surgery, Shanghai Tongji Hospital, School of Medicine, Tongji University, Xincun Rd. 389, 200065 Shanghai, P.R. China

**Keywords:** Circular RNA, Esophageal squamous cell carcinoma, Cisplatin resistance, CPEB4, Mcl-1

## Abstract

**Background:**

Cisplatin-based chemotherapy is a mainstay systematic therapy for advanced esophageal squamous cell carcinoma (ESCC), and cisplatin resistance, which is not uncommon, is the major barrier to improving patient outcomes. Circular RNAs (circRNAs) are novel noncoding RNAs that are implicated in cancer progression, but their involvement in modulating cisplatin responsiveness in ESCC remains unknown.

**Methods:**

Bioinformatics analysis was used to profile and identify the circRNAs involved in cisplatin responsiveness in ESCC. The chemosensitive role of cDOPEY2 was confirmed both in vitro and in vivo. The molecular mechanism of cDOPEY2 was investigated by mass spectrometry, immunoprecipitation, and ubiquitination analyses.

**Results:**

We report that a novel circRNA (cDOPYE2, hsa_circ_0008078) was markedly downregulated in cisplatin-resistant ESCC cells (ESCC-CR) compared with parental chemosensitive cells. Re-expression of cDOPEY2 substantially enhanced the cell-killing ability of cisplatin by augmenting the apoptotic process in ESCC-CR cells, which was achieved by decreasing the abundance of the antiapoptotic protein Mcl-1. Mechanistically, we showed that cDOPEY2 acted as a protein scaffold to enhance the interaction between the cytoplasmic polyadenylation element binding protein (CPEB4) and the E3 ligase TRIM25, which in turn facilitated the ubiquitination and degradation of CPEB4. The increased Mcl-1 expression in ESCC-CR cells was dependent on the binding of CPEB4 to its untranslated mRNA, and depletion of CPEB4 mediated by cDOPEY2 reversed this effect. Rescue experiments confirmed that the critical role of cDOPEY2 in maintaining cisplatin sensitivity was dependent on the depletion of CEPB4 and its downstream target Mcl-1. Clinical and in vivo data further corroborated the significant relevance of cDOPEY2 to cisplatin responsiveness in ESCC.

**Conclusions:**

We provide evidence that cDOPEY2 inhibits CPEB4-mediated Mcl-1 translation by promoting the ubiquitination and degradation of CPEB4 to alleviate cisplatin resistance, indicating that cDOPEY2 may serve as a valuable biomarker and potential therapeutic target in ESCC.

**Supplementary Information:**

The online version contains supplementary material available at 10.1186/s13046-021-02149-5.

## Background

Esophageal cancer (EC) is one of the most commonly diagnosed malignancies and the leading cause of cancer-related death worldwide [1]. Esophageal squamous cell carcinoma (ESCC) is the most common histological subtype of EC [2]. Most ESCC patients present with a locally advanced stage at the time of diagnosis, and the treatment of choice for these cases is neoadjuvant chemotherapy and radiotherapy followed by radical surgery [3]. However, the outcomes of patients treated with this modality remain largely unsatisfactory, and most patients die due to tumor progression [4]. Cisplatin is one of the most widely used and effective chemotherapeutic agents for patients with ESCC [5]. Unfortunately, tumors initially respond adequately to cisplatin but rapidly acquire chemoresistance during long-term exposure, and patients thereby relapse or develop metastatic disease [6]. Substantial effort has been dedicated to elucidating the mechanisms underlying cisplatin resistance, including decreased drug accumulation, reduced apoptotic responses and increased tolerance to DNA damage [7, 8]. However, knowledge on strategies to overcome such limitations and the identification of biomarkers capable of predicting cisplatin responsiveness remain limited.

Circular RNAs (circRNAs) are a class of highly stable noncoding RNAs with a covalently closed single-stranded loop conformation that originate from the back-splicing of premRNA exons [9]. Previous studies have shown that circRNAs play functional roles in fundamental processes by sponging miRNAs, modulating gene expression at the transcription or splicing level, serving as protein scaffolds or even directly encoding functional polypeptides [10, 11]. The landscape and actions of circRNAs have been widely reported in ESCC [12]. To date, however, whether circRNAs are implicated in the acquisition of cisplatin resistance and the underlying mechanism remain unclear.

Mcl-1 (myeloid cell leukemia cell differentiation protein-1) belongs to the antiapoptotic Bcl-2 family of proteins, which can promote cell survival and inhibit apoptosis by inactivating their proapoptotic Bcl-2 family counterparts [7]. Increasing evidence suggests that sustained Mcl-1 expression is a key determinant for the evasion of chemotherapeutic reagent-induced cancer cell death. Among all Bcl-2 antiapoptotic family members, only Mcl-1 has been found to be upregulated in the majority of cisplatin-resistant cells [13]. Targeting Mcl-1 in ESCC also restores its chemosensitivity and inhibits its progression [14, 15]. However, the regulatory mechanism of Mcl-1 in ESCC remains elusive.

In this study, we demonstrate that a specific circRNA, cDOPEY2, is markedly downregulated in ESCC cells acquiring cisplatin resistance. Clinically, decreased cDOPEY2 predicts unfavorable overall survival and low cisplatin responsiveness. Functional studies indicated that the re-expression of cDOPEY2 potently attenuates cisplatin resistance in ESCC cells. Further mechanical investigations revealed a critical role for cDOPEY2 in antagonizing the expression of the antiapoptotic protein Mcl-1 in ESCC cells. We reveal, for the first time, that cDOPEY2 enhances the TRIM25-mediated ubiquitination and degradation of the cytoplasmic polyadenylation element binding protein CPEB4, an exemplary key posttranscriptional regulator that we found to promote Mcl-1 translation and expression. Therefore, we herein identify cDOPEY2 as an emerging circRNA that is functionally important for ESCC chemosensitivity.

## Materials and methods

### Clinical samples

#### Cohort I

Human ESCC tissues and paired-adjacent nontumorous tissues (PANTs) were obtained from 48 patients undergoing surgery at Shanghai Tongji Hospital from 2012 to 2014. The collected samples were preserved in liquid nitrogen for further quantitative polymerase chain reaction (qPCR) analyses.

#### Cohort II

A total of 56 ESCC patients treated with cisplatin-based chemotherapy between 2011 and 2015 at Shanghai Tongji Hospital were enrolled in this study.

This study was approved by the Medical Ethics Committee of Shanghai Tongji Hospital. Written informed consent was obtained from each patient before the study began, and all of the patients were followed up on a regular basis. The detailed clinicopathological characteristics are listed in Tables S1-2.

### Cell culture and reagents

HEK293T cells, human ESCC cell lines (ECA109, TE1, TE10, KYSE30, KYSE150, KYSE410, and EC9706), and human normal esophageal epithelial cell lines (HEEC and Het-1a) were purchased from the Cell Bank of the Chinese Academy of Sciences (Shanghai, China). HEK293T and HEEC cells were cultured in DMEM (Gibco, MA, USA) supplemented with 10 % FBS (Gibco, MA, USA). ECA109, TE1, TE10, KYSE150, and EC9706 cells were cultured in RPMI-1640 medium (Gibco, MA, USA) supplemented with 10 % FBS. KYSE30 and KYSE410 cells were cultured in F12-K medium (Gibco, MA, USA) supplemented with 10 % FBS. Het-1a cells were cultured using BEGMTM BulletKit^TM^ (Lonza, GA, USA). All cell lines were cultured at 37 °C with 5 % CO_2_, authenticated by the short tandem repeat (STR) method, and evaluated for mycoplasma contamination.

### Plasmid construction

For cDOPEY2 overexpression, a fragment containing the EcoRI site, splice acceptor AG, linear ASK1 cDNA, splice donor GT, and BamHI site, in that order, was constructed by chemical gene synthesis (Invitrogen GeneArt^TM^, Invitrogen, MA, USA) and inserted into the PLO5-ciR plasmid with a puromycin selection tag (Geneseed, Guangdong, China). For cDOPEY2 silencing, a short hairpin RNA (shRNA) targeting the junction site of cDOPEY2 was synthesized and subcloned into the shRNA expression vector pLVX-shRNA (TaKaRa Bio, Beijing, China).

Full-length CPEB4 and TRIM25 cDNA sequences were amplified by qPCR using total RNA extracted from HEK293T cells. myc-ubiquitin, myc-TRIM25, and myc-CPEB4 were subcloned into a pRK5 vector with a myc tag, Flag-CPEB4 was subcloned into a pRK5 vector with a Flag tag, and His-ubiquitin was subcloned into a pcDNA3.1/His A vector (Invitrogen, CA, USA) with a His tag. Fragments of truncated CPEB4 and TRIM25 were generated by chemical gene synthesis (Invitrogen GeneArt^TM^), and the pRK5 vector with a myc tag served as the backbone plasmid. The siRNA and shRNA sequences are listed in Table S3.

### RNA extraction and qPCR

Total RNA was extracted using TRIzol (Invitrogen, MA, USA) and then converted into cDNA with a PrimeScript RT Reagent Kit (TaKaRa Bio, Beijing, China). Nuclear and cytoplasmic extraction reagents (NE-PER, Pierce, IL, USA) were employed to isolate fractional RNA in the cytoplasm and nucleus. Real-time qPCR was performed using SYBR Premix Ex Taq (TaKaRa Bio, Beijing, China) as previously described [16]. The expression levels of the RNAs of interest were normalized to those of GAPDH, and the primers used in this study are listed in Table S3.

### Protein isolation and western blotting

Total proteins were extracted using RIPA lysis buffer (Solarbio, Beijing, China) according to the manufacturer’s protocol, and the concentration of extracted proteins was measured by the bicinchoninic acid (BCA) method (Solarbio, Beijing, China). Proteins were separated by 10 % SDS–PAGE and then transferred onto PVDF membranes (Invitrogen, MA, USA). Subsequently, the membranes were blocked and incubated with a primary antibody overnight at 4 °C, followed by incubation with a secondary antibody (Abcam, Cambridge, UK) at room temperature for 1 h. The bands were developed by the enhanced chemiluminescence (ECL) method (EpiZyme, Shanghai, China). The antibodies used in this study are listed in Table S4.

### Cell viability and apoptosis assay

For the cell viability assay, 1 × 10^3^ cells were seeded in 100 µL of complete culture medium in 96-well plates for various amounts of time. The cell counting kit-8 assay (Dojindo Laboratories, Kumamoto, Japan) was conducted to assess cell viability according to the manufacturer’s instructions. For the apoptosis assay, 1 × 10^5^ cells were double-labeled with Annexin V-FITC/PI according to the manufacturer’s protocol (BD Bioscience, NJ, USA), and the apoptotic rate was determined by flow cytometry (Beckman Coulter, CA, USA) and analyzed using FlowJo software (TreeStar, OR, USA).

### Clonogenic formation assay

Briefly, equal numbers of ESCC cells were plated in 6-well plates at a density of 400 cells per well and allowed to adhere overnight. Then, the cells in each well were treated with cisplatin at concentrations of 5, 10, 15, 20, and 25 µg/mL. After 14 days of culture, clones were stained with crystal violet, and the survival fraction was calculated as follows: (number of colonies formed/number of cells plated)_cisplatin−treated_/(number of colonies formed/number of cells plated)_control_.

## Ubiquitination assay

Cells transfected with various constructs were cotransfected with FLAG-CPEB4 and Myc-Ub or His-Ub, treated with MG132, and then subjected to lysis using RIPA lysis buffer. Ubiquitination of CPEB4 was detected by IP with an antibody against the Flag tag and then by western blotting with an anti-Ub antibody (ab134953, Abcam).

### Polysome profiling analysis

ESCC cells were lysed in hypotonic buffer (5 mM Tris-Cl (pH 7.5), 2.5 mM MgCl2, 1.5 mM KCl, 1× protease inhibitor cocktail, 0.5 % Triton X-100, 0.1 mg/mL CHX and 0.5 % sodium deoxycholate) at 4 °C for 20 min, followed by centrifugation at 16,000×g for 7 min. Then, the lysate supernatant was collected and carefully added to the sucrose gradient solution. The samples were subjected to ultracentrifugation at 20,000×g for 2 h at 4 °C, and the sucrose gradient was then collected from top to bottom. RNA was recovered from each fraction with TRIzol, and the mRNA expression of Mcl-1 in each fraction was determined by qPCR.

### Liquid chromatography coupled tandem mass spectrometry (LC-MS/MS) analysis

The MS analysis procedures have been described in detail by Xu et al.[17]. In brief, proteins separated by SDS-PAGE were digested using sequencing-grade trypsin (Promega, WI, USA), and the digested peptides were desalted, sequentially dissolved and eluted in Buffer A/B (formic acid/acetonitrile), and separated with an HPLC chromatography system (Easy-nLC1000, Thermo Fisher, MA, USA). Then, the samples were analyzed with a tandem QExactive mass spectrometry system (Thermo Fisher Scientific, MA, USA) and data-dependent MS/MS acquisition (higher-energy collision dissociation with scans (50 to 3000 m/z) using FTMS at a mass resolution of 120,000), selecting the ten most abundant precursor ions at 35 % collision energy at a mass resolution of 30,000 with a dynamic exclusion duration of 15.0 s. The data were analyzed with Proteome Discoverer software (Thermo Fisher Scientific, MA, USA). The signal of each protein was obtained from three technical replicates.

### Immunoprecipitation (IP) and RNA immunoprecipitation (RIP)

For IP, the extracted proteins were first precleared using protein A/G agarose beads. Then, a negative control IgG or primary antibody against the indicated protein was added to the lysates, which were incubated with protein A/G agarose beads at 4 °C overnight. The beads were collected and washed with IP buffer, and the protein complexes were harvested in SDS loading buffer (Solarbio, Beijing, China) and further analyzed by western blotting.

For RIP, a Magna RIP RNA-Binding Protein Immunoprecipitation Kit (Millipore, MA, USA) was used according to the manufacturer’s instructions. Briefly, cells were lysed in RIP buffer, centrifuged, and incubated with protein A/G agarose beads and primary antibodies at 4 °C overnight. Then, the lysates were centrifuged and washed, and the immune complexes were recovered. The bead-bound proteins were further analyzed by western blotting. The isolated RNA was quantified by qPCR and normalized to the input.

### Luciferase assay

The promoter region of DOPEY2 was synthesized and subcloned into the pGL3 vector (Promega, WI, USA). Cisplatin-resistant and parental ESCC cells were seeded in 24-well plates and transfected with reporter plasmids using Lipofectamine 3000 (Invitrogen, CA, USA). After 48 h, the relative luciferase activity was measured with a Dual Luciferase Reporter Assay System (Promega, WI, USA); Renilla luciferase activity was used as an internal control.

### RNA pulldown

Cells were first lysed in lysis buffer (Beyotime, Beijing, China) and then precleared using streptavidin-coated magnetic beads (Invitrogen, MA, USA). Subsequently, the lysate was incubated with streptavidin-coated magnetic beads immobilized with probes complementary to CPEB4, Mcl-1, or the splice junction of cDOPEY2 overnight at 4 °C. The biotin-coupled RNA complexes were magnetically separated and washed. The beads were eluted with SDS buffer (Solarbio, Beijing, China) for western blot analysis and with nonionic water for MS analysis.

### Bioinformatics

Data from a Gene Expression Omnibus (GEO) data set (GSE131969) were used to analyze the expression of circRNAs in ESCC samples. The annotation of circRNAs was based on curated data in circBase. Raw data were collected from these data sets and manipulated, analyzed, and presented with R 3.6.0 and GraphPad Prism 7.0. All codes supporting the conclusions of the study are available from the corresponding author upon reasonable request.

### Xenograft model

Three- to four-week-old female BALB/c nude mice were purchased from SLAC (Shanghai, China) and housed under pathogen-free conditions. ECA109 cells (5 × 10^6^) and their cisplatin-resistant counterparts were subcutaneously injected into the dorsal flank of each mouse, and tumor volumes were measured every 5 days with electronic calipers. Thirty days after inoculation, the animals were euthanized, and tumor samples were collected. Subsequently, the tissues were fixed in 4 % paraformaldehyde for IHC analysis.

### Statistical analysis

The data are presented as the mean ± SD, and data analysis was conducted using GraphPad Prism 7.0. Unpaired or paired 2-tailed Student’s t-tests were conducted for comparisons between 2 groups, and one-way ANOVA with Tukey’s post hoc test was performed to compare more than 2 groups. Correlations between the expression levels of different factors were assessed by Spearman correlation coefficient analyses. The survival data were analyzed using the log-rank test and are presented as Kaplan-Meier survival curves. All data were assessed preliminarily to ensure that they met the assumptions of the statistical analysis (e.g., normal distribution, adequate statistical power, and homogeneity of variance). All experiments were repeated at least three times, and differences with *P* < 0.05 were considered significant.

### Methods and materials are described in full in the Supplementary files

## Results

### Identification of a novel circRNA (cDOPEY2) downregulated in cisplatin-resistant ESCC cells and its characterization

**Fig. 1:**
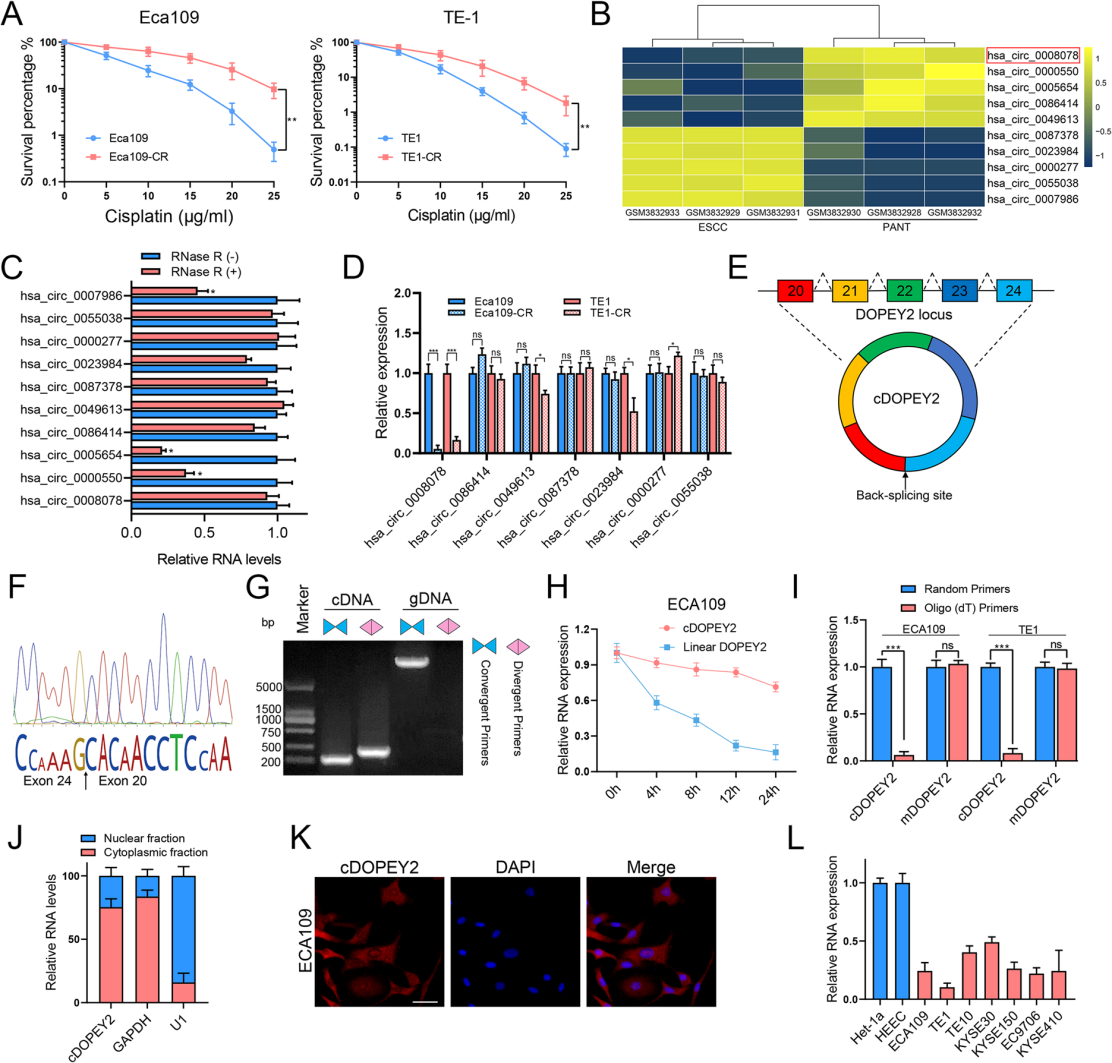
Identification and characterization of a cisplatin sensitivity-associated circRNA (cDOPEY2). **A** The cisplatin sensitivity of two cisplatin-resistant ESCC cell lines (ECA109-CR and TE1-CR) was determined by a clonogenic formation assay. **B** A heatmap showing the top 5 most upregulated circRNAs and 5 most downregulated circRNAs in ESCC tissues and paired adjacent nontumorous tissues (PANTs) in GSE131969. cDOPEY2 (hsa_circ_0008078) is indicated by the red box. **C** qPCR analysis of the RNA levels of the indicated circRNAs with or without RNase R treatment. **D** qPCR analysis of the RNA levels of the indicated circRNAs in cisplatin-resistant cells and their corresponding parental cells. **E** A schematic diagram showing that cDOPEY2 was formed by the back-splicing of linear cDOPEY2 between the 20th and 24th exons. **F** The cDOPEY2 junction site was identified by Sanger sequencing. **G** qPCR analysis of cDOPEY2 and its linear counterpart DOPEY2 in complementary DNA (cDNA) and genomic DNA (gDNA). **H** Time-course qPCR analysis of cDOPEY2 and its linear counterpart DOPEY2 in ECA109 cells treated with actinomycin D (5 µg/mL). **I** qPCR amplification of cDOPEY2 and its linear counterpart DOPEY2 using random hexamer primers and oligo (dT) primers. **J** Subcellular qPCR analysis showing that cDOPEY2 was mainly localized in the cytoplasm. GAPDH and U1 were applied as positive controls in the cytoplasm and nucleus, respectively. **K** RNA fluorescence in situ hybridization analysis revealed the subcellular localization of cDOPEY2. Scale bar, 20 μm. **L** Relative expression of cDOPEY2 in a set of ESCC cell lines and normal esophageal epithelial cells (Het-1a and HEEC). Data are presented as the mean ± SD. *P < 0.05, **P < 0.01, ***P < 0.001. P values were determined using the unpaired Student’s t-test (**A**,** C**,** D**,** H** and **I**)

To explore the molecular mechanism underlying cisplatin resistance in ESCC cells, we initially established two cisplatin-resistant ESCC cell lines (Eca109-CR and TE1-CR) by consecutively culturing the cells in media containing increasing concentrations of cisplatin; their cisplatin resistance was then determined by conducting a clonogenic assay [18]. As shown in Fig. 1 A, both cisplatin-resistant cell lines exhibited a higher survival rate after cisplatin treatment than their parental cells. A circRNA may participate in regulating cisplatin resistance only if it is significantly differentially expressed in ESCC tissues compared with nontumorous tissues. Thus, we analyzed the expression profiles of circRNAs in ESCC from a GEO data set (GSE131969) to identify dysregulated circRNAs (Fig. 1B). Among the 5 most upregulated and 5 most downregulated circRNAs, 7 were shown to be authentic circRNAs based on the results of RNase R treatment (Fig. 1 C). We further validated the expression of these circRNAs in Eca109-CR/Eca109 and TE1-CR/TE1 cells by qRT-PCR, revealing that only hsa_circ_0008078, a circRNA derived from the DOPEY2 gene, was consistently and significantly downregulated in cisplatin-resistant cells compared with their parental cells (Fig. 1D). We thus further investigated the potential involvement of hsa_circ_0008078, named cDOPEY2, in ESCC cell cisplatin resistance.

Annotation from circBase (http://circrna.org) revealed that cDOPEY2 originated from the back-splicing of the DOPEY2 gene between the 20th and 24th exons (Fig. 1E). We next utilized several approaches to characterize the nature of cDOPEY2. We first identified the back-splicing site of cDOPEY2 by Sanger sequencing (Fig. 1 F) and then used divergent primers to amplify cDOPEY2 from cDNA but not from gDNA (Fig. 1G). Next, actinomycin D treatment was performed, confirming that cDOPEY2 was more stable than its parental host gene (Fig. 1 H). Moreover, cDOPEY2 was significantly enriched in samples that were reverse-transcribed using random hexamer primers compared with that in samples reverse-transcribed with oligo (dT) primers (Fig. 1I). Ultimately, qPCR of subcellular fractions and fluorescence in situ hybridization (FISH) analyses showed that cDOPEY2 was mainly located in the cytoplasm (Fig. 1 J-K).

Moreover, assessment of cDOPEY2 expression in ESCC cell lines revealed that it was not only significantly downregulated in cisplatin-resistant cells but also markedly decreased in ESCC cell lines compared with the normal esophageal squamous epithelial cell lines HECC and Het-1a (Fig. 1 L).

Taken together, these results revealed a novel circular RNA (cDOPEY2) that was downregulated in cisplatin-resistant ESCC cells, indicating a potential suppressive effect of cDOPEY2 in mediating cisplatin resistance.

### cDOPEY2 attenuates cisplatin resistance by enhancing cisplatin-induced apoptosis in ESCC

**Fig. 2:**
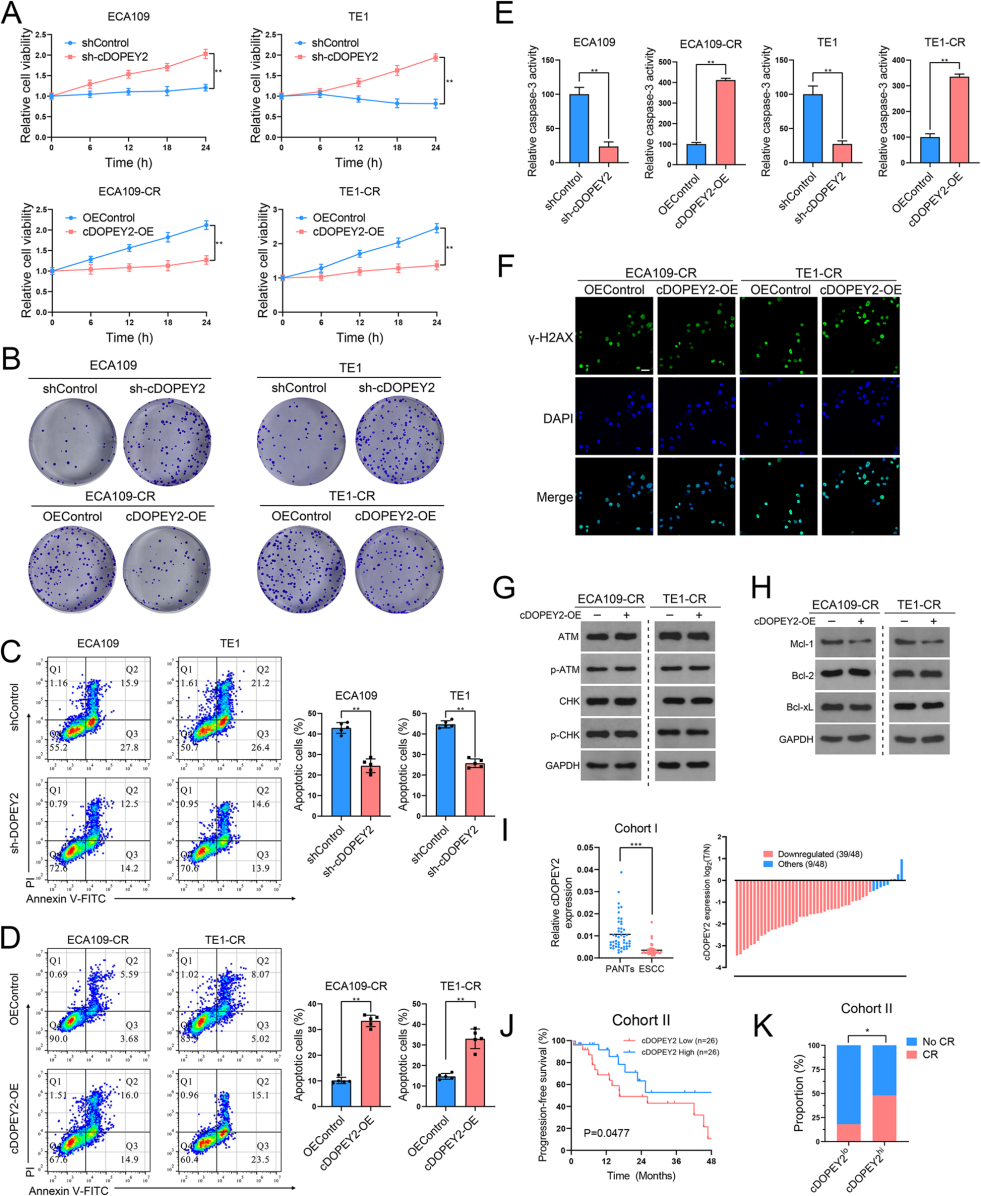
cDOPEY2 attenuates cisplatin resistance in ESCC cells. **A** The viability of the indicated cells with cDOPEY2 overexpression or knockdown after treatment with cisplatin (10 µg/mL) was measured by the CCK-8 method. **B** The impact of cDOPEY2 on ESCC cells treated with cisplatin was determined by a clonogenic assay. **C-D** The apoptotic rate of the indicated cells with cDOPEY2 overexpression or knockdown after treatment with cisplatin (10 µg/mL) for 24 h was determined by Annexin V-FITC/PI FACS analysis. **E** Relative caspase-3 activity in the indicated ESCC cells 24 h after cisplatin treatment at a dose of 10 µg/mL. **F** Representative γ-H2AX staining images of the indicated ESCC cells 24 h after cisplatin treatment at a dose of 10 µg/mL. Scale bars: 20 μm. **G-H** Western blot analysis of the indicated proteins in cDOPEY2-overexpressing and cDOPEY2-silenced cells 24 h after cisplatin treatment at a dose of 10 µg/mL. **I** Relative expression of cDOPEY2 in 48 paired ESCC samples in cohort (I) GAPDH was used as an internal control. **J** Kaplan-Meier (**K**-**M**) plot showing the relationship between cDOPEY2 expression and patient progression-free survival (PFS) in cohort (II) **K** Bar graph showing that cDOPEY2 expression was positively correlated with the complete response rate (CR) among patients receiving cisplatin-based chemotherapy in Cohort II. Data are presented as the mean ± SD. *P < 0.05, **P < 0.01, ***P < 0.001. P values were determined with the unpaired Student’s t-test (**A**,** C**,** D** and **E**), paired Student’s t-test (**I**), log-rank test (**J**), and Fisher’s exact-test (**K**)

We next investigated the impact of cDOPEY2 on cisplatin resistance in ESCC by overexpressing cDOPEY2 in cisplatin-resistant cells and silencing cDOPEY2 in cisplatin-sensitive cells using lentiviral-based constructs (Fig. S1A-B). Cell viability and colony formation assays revealed that high cDOPEY2 expression markedly sensitized cisplatin-resistant ESCC cells to cisplatin treatment, while depletion of cDOPEY2 exerted the opposite effects on parental ESCC cells (Fig. 2 A-B), suggesting an important role of cDOPEY2 in alleviating cisplatin resistance. We further assessed cisplatin-induced apoptosis by Annexin-V/PI staining, clearly showing that cDOPEY2 overexpression in cisplatin-resistant cells boosted cisplatin-mediated apoptosis (Fig. 2 C-D). Consistently, caspase-3 activity was enhanced by cDOPEY2 in cells treated with cisplatin (Fig. 2E). Because cisplatin kills tumor cells by inducing replication fork arrest and subsequently causing replication stress [19], we next explored whether cDOPEY2 impacted DNA damage in tumor cells by performing γ-H2AX staining. Although γ-H2AX staining was markedly decreased in cisplatin-resistant cells compared with parental cells, cDOPEY2 did not impact cisplatin-induced DNA damage (Fig. 2 F). We obtained similar results by determining the activation of the DNA damage repair (DDR) pathway (Fig. 2G). The abundances of antiapoptotic regulatory proteins of the Bcl-2 family were further determined by western blotting, revealing that cDOPEY2 upregulation significantly decreased the expression of antiapoptotic Mc1-1. However, the other antiapoptotic proteins, Bcl-xL and Bcl-2, were unchanged following cDOPEY2 alteration (Fig. 2 H). Together, these results indicated that cDOPEY2 played a vital role in alleviating ESCC resistance to cisplatin and that this effect was potentially exerted via the exaggeration of the cisplatin-induced apoptotic process.

We next explored the clinical significance of cDOPEY2 by examining its expression in clinical samples from ESCC patients. Confirming the previous microarray results, cDOPEY2 was significantly downregulated in 81 % (39 of 48) of the ESCC samples (cohort I, containing 48 pairs of tumor/nontumor samples) compared with the adjacent nontumorous tissues (Fig. 2I). In a second cohort of patients with advanced ESCC receiving cisplatin treatment, low cDOPEY2 expression in tumor samples was significantly associated with a lower response rate and worse progression-free survival (PFS) than those of patients with high cDOPEY2 tumor expression levels (Fig. 2 J-K). These findings indicate that cDOPEY2 has potential as a suitable marker for predicting the efficacy of cisplatin responsiveness in ESCC patients.

## cDOPEY2 physically interacts with the RNA-binding protein (RBP) CPEB4

We further dissected the mechanism by which cDOPEY2 alleviates cisplatin resistance in ESCC cells. First, cDOPEY2 was determined to be mainly localized in the cytoplasm, as shown in Fig. 1 J-K. However, AGO2 RIP showed that cDOPEY2 did not coimmunoprecipitate with AGO2, indicating that it likely did not act as a competitive RNA (Fig. S2A). Second, no potential open read frame (ORF) or internal ribosomal entry site (IRES) sequences were found in cDOPEY2. Third, cDOPEY2 was unable to influence the expression of its host gene, DOPEY2 (Fig. S2B). Taking the above observations into consideration, we hypothesized that cDOPEY2 functions by acting as a protein scaffold in the cytoplasm.

**Fig. 3:**
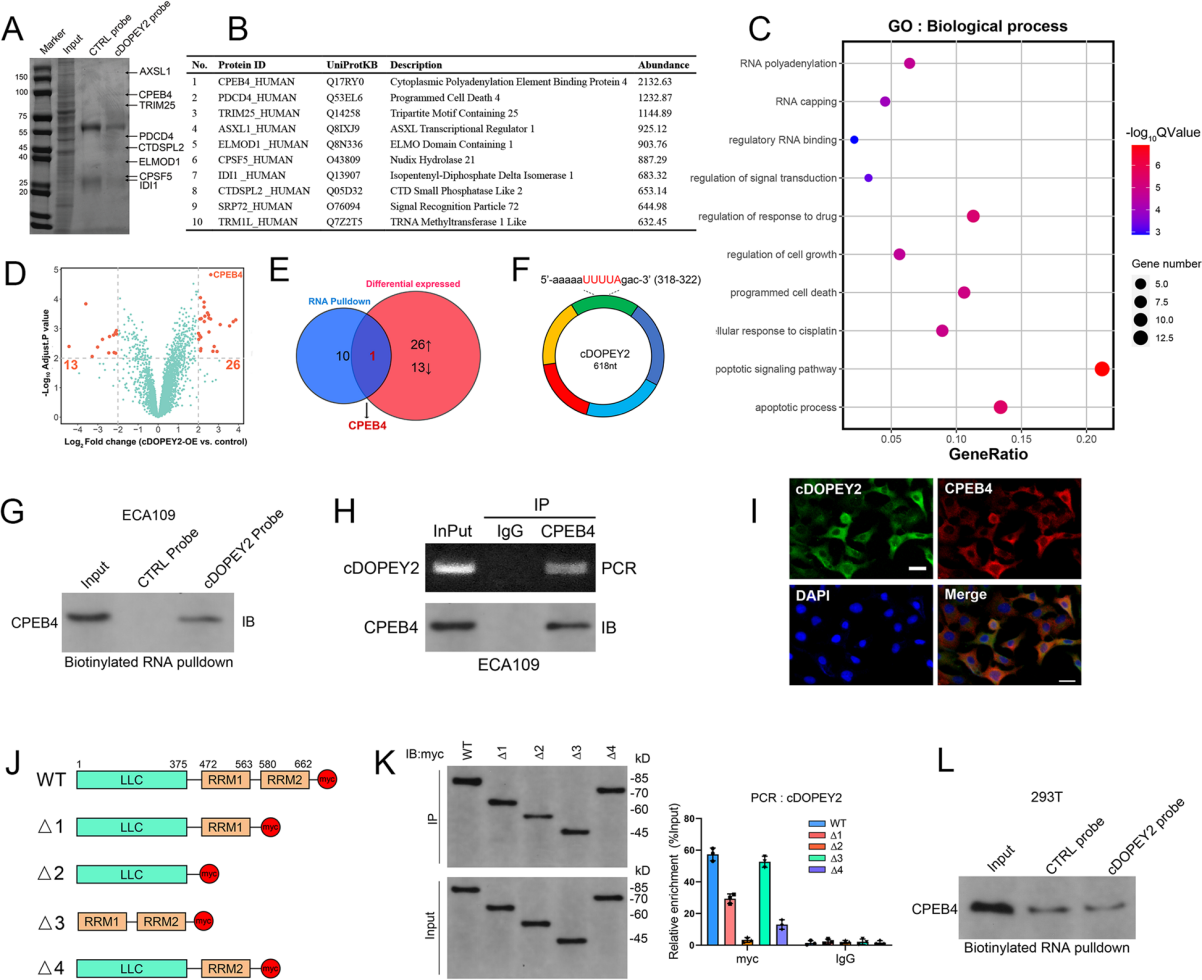
cDOPEY2 physically interacts with CPEB4 protein. **A** Identification of cDOPEY2-protein complexes pulled down by a probe targeting the splicing junction of cDOPEY2 with a protein isolated from ECA109 cells. The arrows indicate the band corresponding to the top-hit protein stained with Coomassie Blue. **B** The top-hit protein enriched with cDOPEY2 as determined by MS analysis. **C** Bubble plot of the top 10 BP terms identified by the GO enrichment analysis of proteins regulated by cDOPEY2 in ECA109 cells. **D** Volcano plot illustrating the dysregulated proteins of interest (fold change > 4 and *P* < 0.01) in the cDOPEY2-overexpressing group compared with controls in ECA109-CR cells. **E** Venn diagram illustrating the overlapping proteins that were both enriched and regulated by cDOPEY2 as determined by MS analysis. **F** A schematic diagram showing the CPEB4 binding motif (UUUUA) of cDOPEY2 at 317-322 nt. **G** A biotinylated probe pulldown assay was performed to detect the interaction between CPEB4 and cDOPEY2 in ECA109 cells. **H** RIP assays were performed to detect the interaction between CPEB4 and cDOPEY2 in ECA109 cells. Bottom, IP efficiency of the CPEB4 antibody. Top, the abundance of CPEB4-associated cDOPEY2 was analyzed by qPCR and normalized to the input control. An IgG antibody was used as a negative control. **I** FISH immunofluorescence showing the colocalization of cDOPEY2 and CPEB4. Scale bars: 20 μm. **J** A schematic diagram illustrating the construction of myc-tagged CEPB4 with different truncations. **K** Left panel: western blotting was performed to assess the IP efficiency of the myc antibody in ECA109 cells with plasmids encoding myc-tagged WT or truncated CPEB4. Right panel: relative enrichment of cDOPEY2 in ECA109 cells with plasmids encoding myc-tagged WT or truncated CPEB4 as determined by qPCR. **L** A biotinylated probe pulldown assay was performed to detect the interaction between CPEB4 and mutant cDOPEY2 in 293T cells

To test our hypothesis, we first conducted an RNA pulldown assay by incubating the extracts of Eca109 cells with a biotinylated probe specifically targeting the junction site of cDOPEY2. The coisolated proteins were separated by SDS-PAGE, stained with Coomassie blue, identified, and analyzed by mass spectrometry (MS) (Fig. 3 A-B). We further performed MS analysis of extracts from cDOPEY2-overexpressing Eca109-CR cells, and compared with control cells, a total of 39 dysregulated proteins were identified (Fig. 3D). Gene ontology (GO) analysis showed that these proteins were significantly correlated with responses to drugs and with the regulation of apoptosis (Fig. 3 C). Among the top 10 most abundant proteins enriched with cDOPEY2, only CPEB4 was significantly regulated by cDOPEY2 (Fig. 3E). CPEB4 is a cytoplasmic RBP that maintains the translation of regulating mRNAs, and importantly, cDOPEY2 harbors a binding sequence (UUUUA) for CPEB4 [20] (Fig. 3 F). Moreover, CPEB4 exhibited the most significantly differential expression following cDOPEY2 overexpression (Fig. 3D). These results strongly suggested that CPEB4 not only bound to but was also regulated by cDOPEY2. Thus, we confirmed the interaction between cDOPEY2 and CPEB4 using RNA pulldown and RNA immunoprecipitation (RIP) assays (Fig. 3G-H). In addition, the RNA FISH immunofluorescence assay revealed that cDOPEY2 colocalized with the CPEB4 protein in the cytoplasm (Fig. 3I).

The RNA recognition motifs (RRMs) of CPEB4 are essential for the interaction between CPEB4 and RNAs containing the UUUUA sequence [20]. To further elucidate the binding mechanism between CPEB4 and cDOPEY2, we constructed several truncated fragments of CPEB4 lacking RRM1, RRM2, or both (Fig. 3 J). RIP demonstrated that the binding of CPEB4 with cDOPEY2 required both RRMs, as deletion of either RRM1 or RRM2 reduced the level of binding between cDOPEY2 and CPEB4, and deletion of both RRMs simultaneously completely abrogated its ability to bind cDOPEY2 (Fig. 3 K). To confirm whether cDOPEY2 interacted with CPEB4 via its UUUUA sequence, we mutated the cDOPEY2 UUUUA sequence to GACCG, which significantly reduced the interaction between CPEB4 and cDOPEY2 (Fig. 3 L). These data confirmed that CPEB4 physically bound with cDOPEY2.

### CPEB4 is an oncogene that augments cisplatin resistance in ESCC cells and is targeted by cDOPEY2

**Fig. 4:**
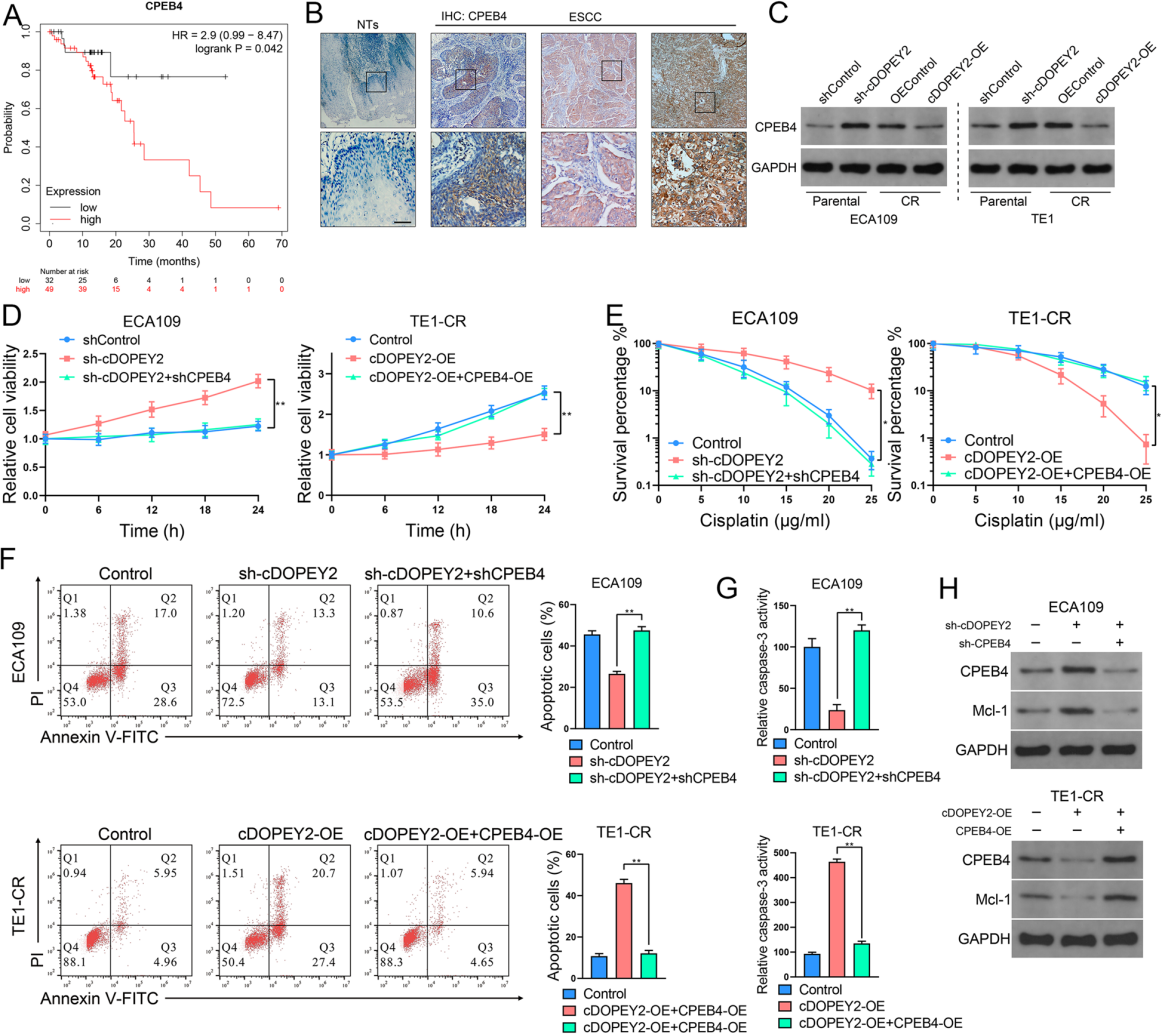
cDOPEY2 functions by suppressing CPEB4. **A** K-M plot showing the relationship between cDOPEY2 levels and patient overall survival (OS) in a TCGA data set. **B** Representative IHC staining images of CPEB4 in ESCC tissues and nontumorous tissues (NTs). Scale bars: 100 μm. **C** Western blotting was performed to detect CPEB4 in the indicated cells with cDOPEY2 overexpression or knockdown. **D** The time-course viability of the indicated cells after treatment with cisplatin (10 µg/mL) was determined by the CCK-8 method. **E** Clonogenic assay of the indicated cells treated with increasing doses of cisplatin. **F** The apoptotic rate of the indicated cells 24 h after treatment with cisplatin (10 µg/mL) was determined by Annexin V-FITC/PI FACS analysis. **G** Relative caspase-3 activity in the indicated ESCC cells 24 h after cisplatin treatment at a dose of 10 µg/mL. **H** Western blotting was performed to detect CPEB4 and Mcl-1 in the indicated cells. Data are presented as the mean ± SD. *P < 0.05, **P < 0.01, ***P < 0.001. P values were determined by one-way ANOVA with Tukey’s post hoc test (**D**,** E**,** F** and **G**) and log-rank test (**A**)

CPEB4 has a well-characterized role in oncogenesis [21, 22], and survival analysis of a TCGA data set confirmed the oncogenic role of CPEB4 in ESCC (Fig. 4 A). Moreover, the CEPB4 protein levels were substantially higher in ESCC samples than in adjacent nontumorous tissues (Fig. 4B). To determine whether the chemosensitive effect of cDOPEY2 was mediated by the depletion of CPEB4, we first examined CPEB4 expression in cisplatin-resistant cells. Western blotting showed that CPEB4 expression was significantly increased in cisplatin-resistant cells compared with their parental cells, while cDOPEY2 overexpression decreased CPEB4 expression, and cDOPEY2 silencing increased the expression of CPEB4 (Fig. 4 C). We further overexpressed CPEB4 in cisplatin-resistant ESCC cells and silenced CPEB4 in parental cells (Fig. S3A-B). cDOPEY2 knockdown in parental Eca109 cells markedly increased cell viability and clonogenic formation ability after cisplatin treatment, whereas cDOPEY2 silencing-induced chemoresistance was almost impaired following CPEB4 knockdown. Conversely, the enhanced sensitivity to cisplatin after cDOPEY2 overexpression in TE1-CR cells was largely rescued by CPEB4 overexpression (Fig. 4D-E). With respect to cisplatin-induced apoptosis, cDOPEY2 depletion led to the acquisition of cisplatin resistance in parental ECA109 cells, as reflected by Annexin-V/PI FACS analysis and caspase-3 activity, and these effects were largely abolished by CPEB4 knockdown. We observed similar results in TE1-CR cells, in which CPEB4 overexpression greatly restored the cisplatin resistance of cells overexpressing cDOPEY2 (Fig. 4 F-G). The increased expression of the antiapoptotic protein Mcl-1 induced by cDOPEY2 depletion was significantly blocked following CPEB4 knockdown, whereas cDOPEY2-suppressed Mcl-1 expression was completely reversed by CPEB4 overexpression (Fig. 4 H). Altogether, these data suggested that cDOPEY2 sensitized ESCC cells to cisplatin by inhibiting CPEB4.

### cDOPEY2 decreases the stability of the CPEB4 protein by enhancing its ubiquitination

**Fig. 5:**
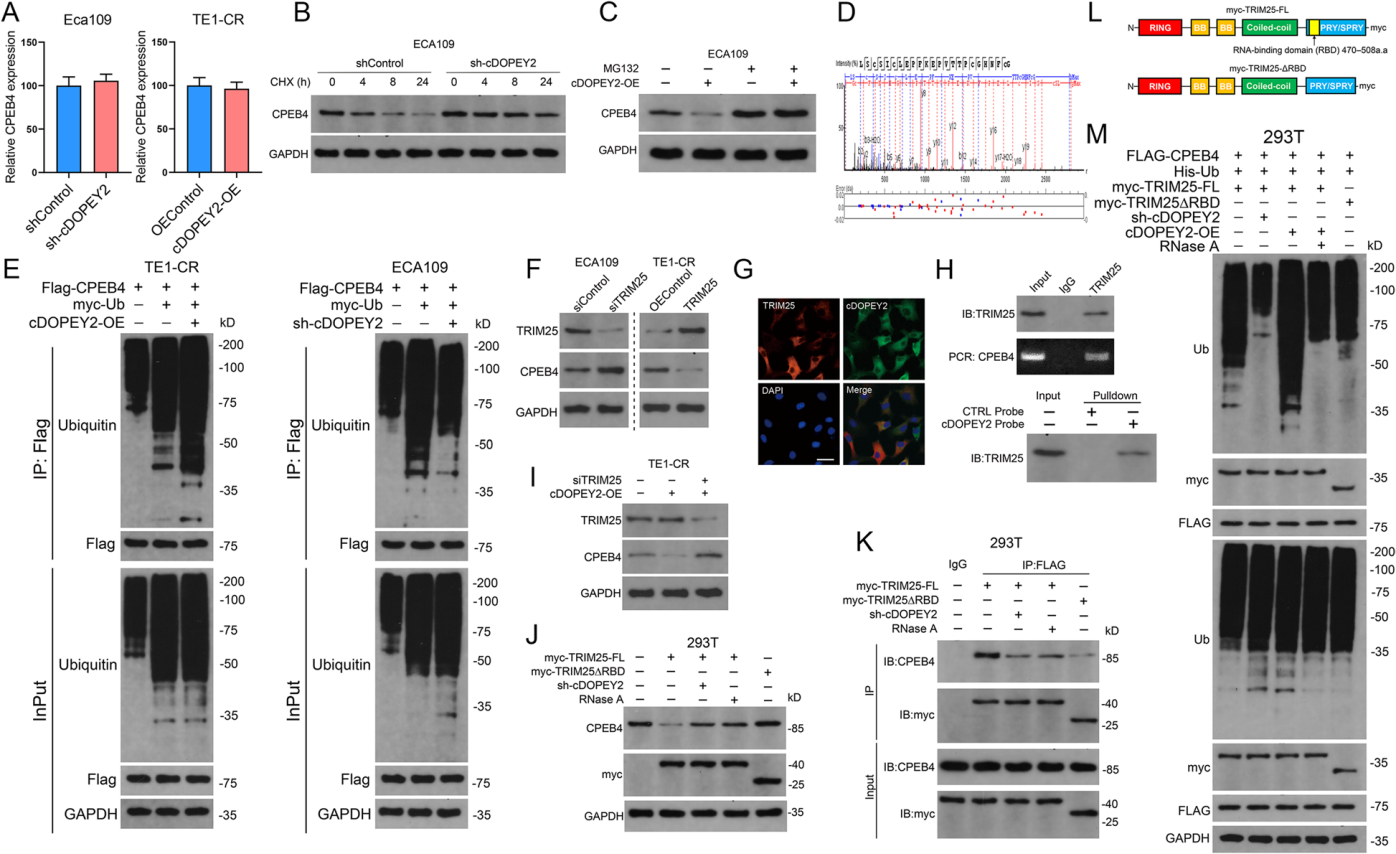
cDOPEY2 serves as a scaffold to enhance the ubiquitination and degradation of CPEB4 mediated by TRIM25. **A** The relative mRNA levels of CPEB4 in the indicated cells as determined by qPCR. **B** Western blot analysis of CPEB4 stability in Eca109 cells with or without cDOPEY2 knockdown after treatment with cycloheximide (CHX, 100 µg/mL). **C** Western blot analysis of CPEB4 stability in control and cDOPEY2-overexpressing Eca109-CR cells treated with or without MG132 (100 µM). **D** TRIM25 was identified as a binding partner of cDOPEY2 by MS. **E** IP analysis of CPEB4 ubiquitination in ESCC cells with cDOPEY2 overexpression or knockdown. **F** Western blot analysis of CPEB4 in ESCC cells transfected with the indicated constructs. **G** FISH immunofluorescence showing the colocalization of cDOPEY2 and TRIM25. Scale bars: 20 μm. **H** RIP (top panel) and biotinylated probe pulldown (bottom panel) assays were performed to detect the interaction between TRIM25 and cDOPEY2. **I-J.** Western blot analysis of CPEB4 in ESCC cells transfected with the indicated constructs. **K** IP analysis of the interaction between CPEB4 and TRIM25 in 293T cells transfected with the indicated constructs. **L** A schematic diagram illustrating the construction of myc-tagged WT and truncated (deletion of the RNA-binding domain) TRIM25. **M** IP analysis of CPEB4 ubiquitination in 293T cells transfected with the indicated constructs. Data are presented as the mean ± SD. P values were determined using the unpaired Student’s t-test (**A**)

As shown in Fig. 4 C, although cDOPEY2 negatively regulated the protein expression of CPEB4, its mRNA expression was not significantly changed by cDOPEY2 (Fig. 5 A). We also found that silencing cDOPEY2 increased the stability of the CPEB4 protein (Fig. 5B). In addition, the reduced protein expression of CPEB4 in cDOPEY2-overexpressing cells was completely reversed by treatment with MG132, a proteasome inhibitor (Fig. 5 C). Thus, we speculated that cDOPEY2 might reduce the stability of CPEB4 by enhancing its ubiquitin/proteasome-dependent degradation. Consistent with our hypothesis, cDOPEY2 overexpression strongly promoted the ubiquitination of CPEB4, while cDOPEY2 knockdown significantly inhibited this phenomenon (Fig. 5E). These data indicated that cDOPEY2 suppressed CPEB4 by inducing its ubiquitination.

### The E3 ligase TRIM25 mediates cDOPEY2 promotion of CPEB4 ubiquitination

We further explored the E3 ligase responsible for the cDOPEY2-mediated ubiquitination of CPEB4. Of all the proteins that coprecipitated with cDOPEY2 as determined by MS, only one E3 ligase, TRIM25, was identified (Fig. 3 A-B, Fig. 5D). Moreover, TRIM25 negatively regulated the expression of CPEB4 at the protein but not at the mRNA level (Fig. 5 F, Fig. S3C-D). The RNA-binding activity of TRIM25 is reportedly essential for the effective ubiquitination of its targeted proteins [23]. RNA FISH immunofluorescence analysis revealed the colocalization of cDOPEY2 with TRIM25 in the cytoplasm (Fig. 5G), and RIP and RNA pulldown assays confirmed the binding between cDOPEY2 and TRIM25 (Fig. 5 H). cDOPEY2 overexpression notably enhanced the effects of TRIM25 on the degradation of CPEB4, while knockdown of TRIM25 largely reversed this cDOPEY2-mediated effect (Fig. 5I). These results indicated that cDOPEY2 might serve as a scaffold to enhance the interaction between TRIM25 and CPEB4. We then explored whether the ubiquitin ligase activity of TRIM25 toward CPEB4 was dependent on cDOPEY2. To achieve this goal, we constructed a myc-tagged TRIM25 fragment in which a portion of the RBD was deleted (ΔRBD, 470–508 aa deletion, Fig. 5 L). IP analysis confirmed that the RBD deletion drastically impaired the ability of TRIM25 to bind CPEB4, while RNase A (an RNA exonuclease blocking the interactions between cDOPEY2 and TRIM25) treatment and cDOPEY2 silencing also weakened the interaction between TRIM25 and CPEB4 (Fig. 5 K). Notably, transfection with TRIM25 lacking an RBD, RNase A treatment and cDOPEY2 knockdown all impaired the effects of TRIM25 on the degradation and ubiquitination of CPEB4 (Fig. 5 J, M). Taken together, our data strongly indicated that cDOPEY2 acted as a scaffold to enhance the interaction between TRIM25 and CPEB4, thereby potentiating TRIM25-dependent ubiquitination and proteasomal degradation.

### CPEB4 upregulates the expression of the antiapoptotic protein Mcl-1 by promoting its translation

**Fig. 6:**
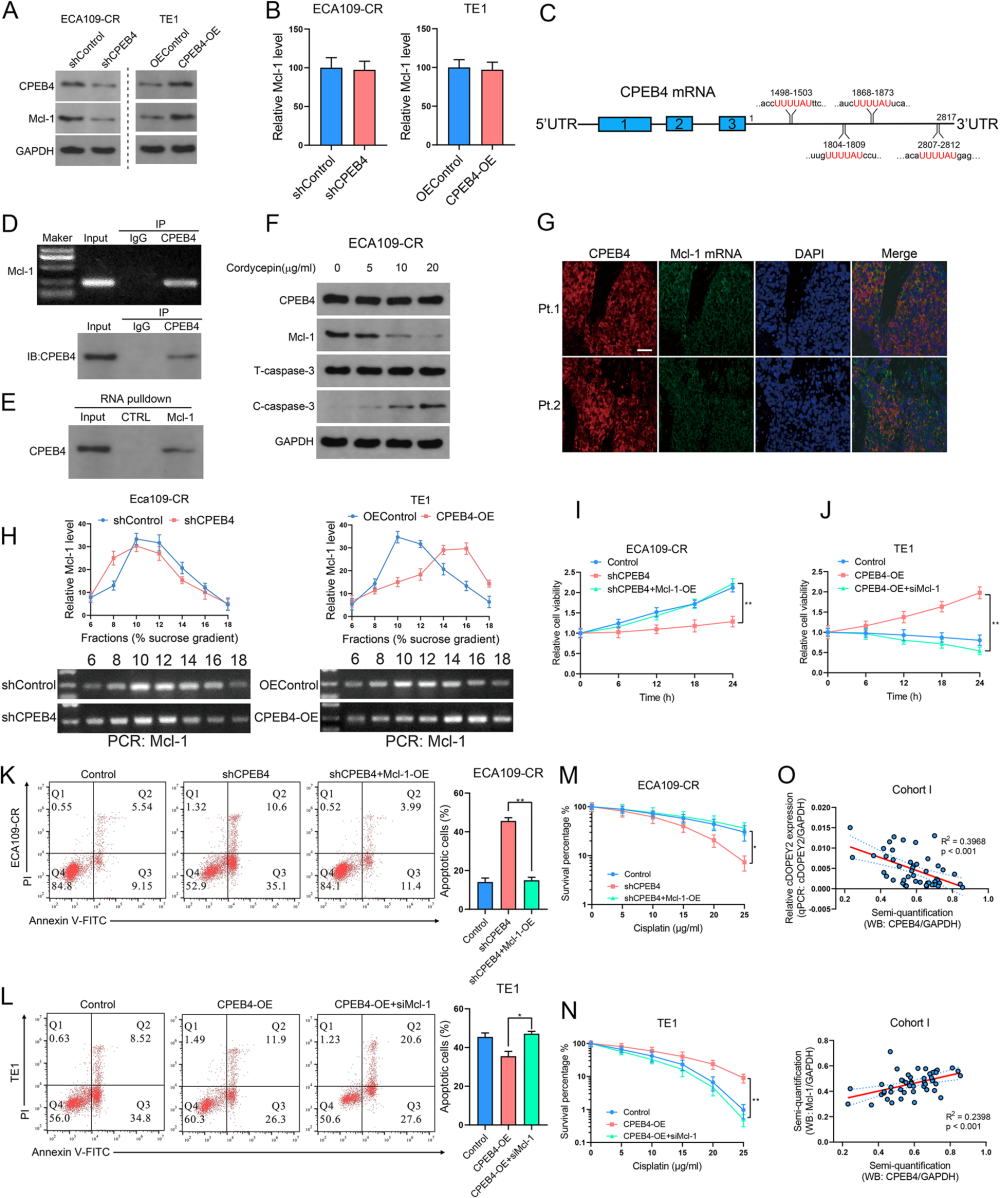
CPEB4 enhances cisplatin resistance by upregulating the expression of the antiapoptotic protein Mcl-1. **A** Western blot analysis of Mcl-1 in ESCC cells with CPEB4 overexpression or knockdown. **B** Relative mRNA level of Mcl-1 in ESCC cells with CPEB4 overexpression or knockdown. **C** A schematic diagram illustrating the putative CPEB4 binding motif in the 3’UTR of Mcl-1. **D** RIP assays were performed to detect the interaction between CPEB4 and Mcl-1 mRNA. Bottom, IP efficiency of the CPEB4 antibody. Top, the abundance of CPEB4-associated Mcl-1 mRNA was analyzed by qPCR and normalized to the input control. An IgG antibody was used as a negative control. **E** A biotinylated probe pulldown assay was performed to detect the interaction between CPEB4 and Mcl-1 mRNA. **F** Western blot analysis of Mcl-1 and caspase-3 expression in ECA109-CR cells in the presence or absence of the polyadenylation inhibitor cordycepin (5, 10 and 20 µg/mL). **G** FISH and IF images of CPEB4 and Mcl-1 mRNA in ESCC samples from cohort II. Scale bars, 100 μm. **H** ESCC cells in which CPEB4 was overexpressed or silenced were sequentially lysed, added to a sucrose gradient, and subjected to ultracentrifugation. The Mcl-1 mRNA levels in each fraction were determined by qPCR. **I** The viability of the indicated cells after treatment with cisplatin (10 µg/mL) was determined by the CCK-8 method. **J** Clonogenic assay of the indicated cells treated with increasing doses of cisplatin. **K** The apoptotic rates of the indicated cells after treatment with cisplatin (10 µg/mL) for 24 h as determined by Annexin V-FITC/PI FACS analysis. **L** The viability of the indicated cells after treatment with cisplatin (10 µg/mL) as determined by the CCK-8 method. **M** Clonogenic assay of the indicated cells treated with increasing doses of cisplatin. **N** The apoptotic rate of the indicated cells after treatment with cisplatin (10 µg/mL) for 24 h was determined by Annexin V-FITC/PI FACS analysis. **O** Correlation between cDOPEY2 RNA expression and CPEB4 protein levels (upper panel) and correlation between Mcl-1 protein abundance and CPEB4 protein levels (bottom panel) in cohort I. Data are presented as the mean ± SD. *P < 0.05, **P < 0.01, ***P < 0.001. P values were determined using the unpaired Student’s t-test (**B**) and one-way ANOVA with Tukey’s post hoc test (**I, J, K, L, M** and **N**). Correlations were determined by Spearman correlation analysis (**O**)

As shown in Fig. 2 H, cDOPEY2 decreased the expression of the antiapoptotic protein Mcl-1. Thus, we wondered whether the inhibitory effect of cDOPEY2 on Mcl-1 was mediated by the decreased expression of its downstream target CPEB4. Importantly, the up- or downregulation of Mcl-1 protein expression following CPEB4 overexpression or silencing was not confirmed at the mRNA level, suggesting that the regulation of Mcl-1 mediated by CPEB4 was modulated at the posttranscriptional level (Fig. 6 A-B). CPEB4 promotes cytoplasmic polyadenylation and the translational activation of mRNAs containing cytoplasmic polyadenylation elements (CPEs) [24]. Notably, because we found several CPEs in the 3’UTR of Mcl-1 mRNA (Fig. 6 C), we speculated that CPEB4 upregulated Mcl-1 expression by promoting its translation. In support of this possibility, RIP analysis confirmed the interaction between CPEB4 and Mcl-1 mRNA (Fig. 6D-E). Moreover, cordycepin (a known inhibitor of CPEB4-mediated polyadenylation) treatment decreased the protein expression of Mc1-1, accompanied by an increase in the expression of cleaved caspase-3, and these effects were dose-dependent (Fig. 6 F). The FISH immunofluorescence assay confirmed that CPEB4 colocalized with Mc1-1 mRNA in ESCC tissues (Fig. 6G).

We performed a ribosome enrichment assay to validate the regulatory effect of CPEB4 on Mcl-1 translation. Extracts of Eca109 cells in which CPEB4 was overexpressed or silenced were subjected to 5–50 % sucrose gradient centrifugation, followed by qPCR analysis of Mcl-1 mRNA expression in each fraction. The distribution of Mcl-1 mRNA shifted from light polysomes to heavy polysomes in CPEB4-overexpressing cells compared with control cells, while CPEB4 silencing shifted the Mc1-1 mRNA-containing fraction from light polysomes to monosomes (Fig. 6 H), confirming that CPEB4 upregulated Mcl-1 expression by promoting its translation. We further validated the involvement of Mcl-1 in CPEB4-mediated cisplatin resistance. Notably, Mcl-1 overexpression significantly rescued the effects of CEPB4 silencing on cisplatin resistance, as determined by cell viability, colony formation, and apoptosis assays (Fig. 6I-K). Moreover, depletion of Mcl-1 completely abolished the enhanced chemoresistance induced by CPEB4 (Fig. 6 K-M). We also analyzed the coexpression pattern between CPEB4 and cDOPEY2 and Mcl-1 in clinical samples and showed that the protein level of CPEB4 was negatively correlated with the cDOPEY2 RNA level but positively associated with the Mcl-1 protein abundance in cohort I (Fig. 6O). Taken together, these results indicated that cDOPEY2 increased cisplatin resistance in ESCC by suppressing CPEB4-induced Mcl-1 translation.

### cDOPEY2 inhibits tumorigenicity and enhances cisplatin sensitivity in ESCC xenograft cells

**Fig. 7:**
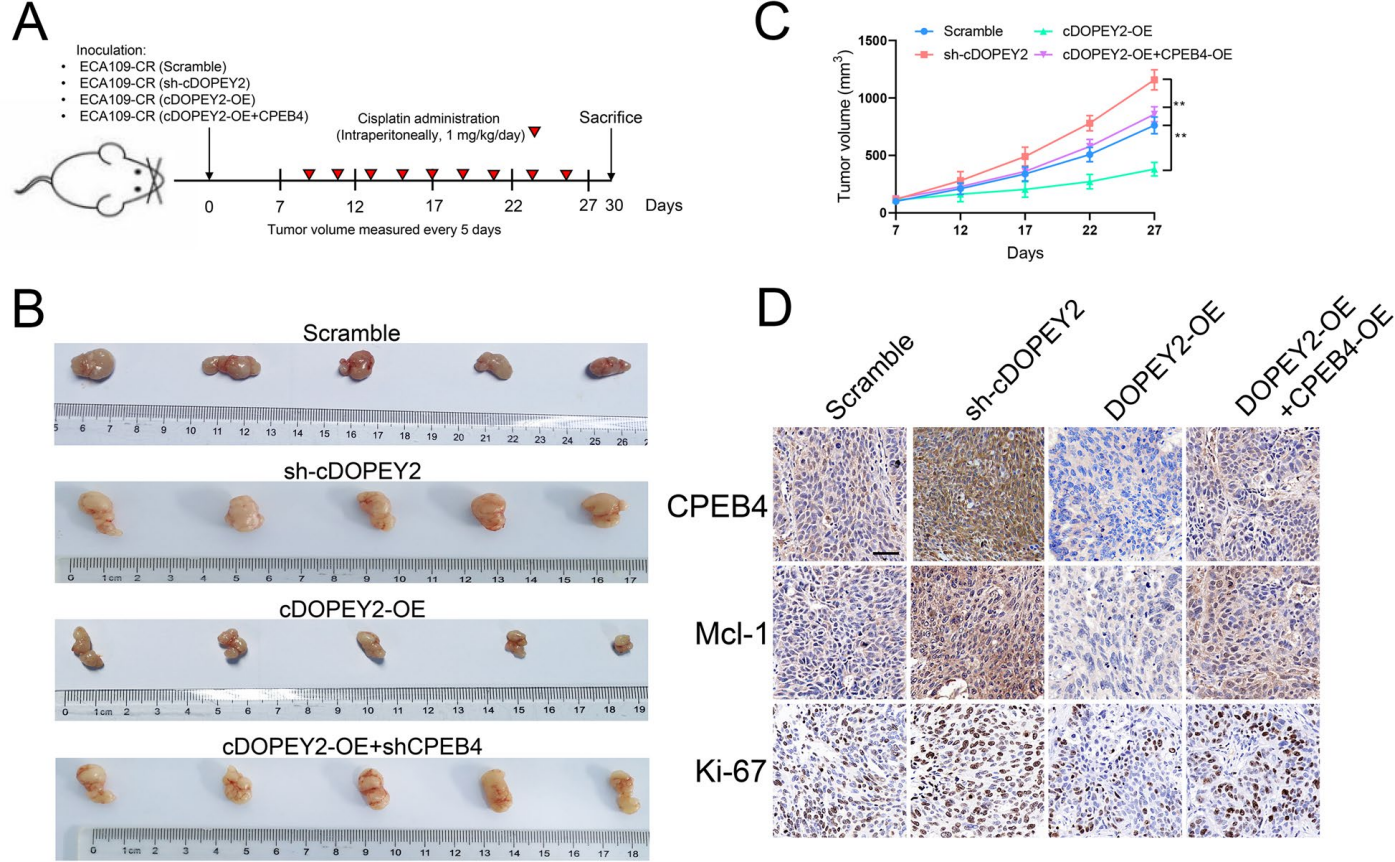
cDOPEY2 attenuates cisplatin resistance in vivo. **A** Schematic diagram demonstrating the grouping and treatment plan of the xenograft model; BALB/c nude mice were inoculated with the indicated cells (5 × 10^6^) and treated with cisplatin (i.p., 1 mg/kg) every two days until the tumor volume exceeded 100 mm^3^. **B** Representative images of the inoculated tumor tissues of each group. **C** Time-course evaluation of the tumor volumes in the indicated groups as measured every 5 days and tumor weights measured after tumors were harvested. **D** IHC staining showing the abundances of CPEB4, Mcl-1, and Ki-67 in the indicated groups. Scale bars, 100 μm. Data are presented as the mean ± SD. *P < 0.05, **P < 0.01, ***P < 0.001. P values were determined by one-way ANOVA with Tukey’s post hoc test

We further verified the biological impact of cDOPEY2 on ESCC in vivo. To achieve this goal, we constructed Eca109-CR cells with cDOPEY2 overexpression, cDOPEY2 knockdown, and cDOPEY2/CPEB4 double overexpression and inoculated the indicated cells into BALB/c nude mice. The tumor-bearing mice were intraperitoneally injected with cisplatin (1 mg/kg) every 2 days (Fig. 7 A). The results demonstrated that silencing cDOPEY2 further enhanced the chemoresistance of Eca109-CR cells. In contrast, nude mice inoculated with cDOPEY2-overexpressing cells exhibited a marked sensitivity to cisplatin resistance, while re-expression of CPEB4 in cDOPEY2-overexpressing cells completely abolished the effects mediated by cDOPEY2 (Fig. 7B-C). Consistent with the in vitro results, immunohistochemistry (IHC) confirmed that the expression of Mcl-1 and CPEB4 was increased in the sh-cDOPEY2 group but decreased in the cDOPEY2 overexpression group compared with the scramble group (Fig. 7D).

These data verified the in vitro findings and supported the potential of cDOPEY2 as a pharmaceutical intervention target to increase the efficacy of cisplatin treatment in patients with advanced ESCC.

**Fig. 8:**
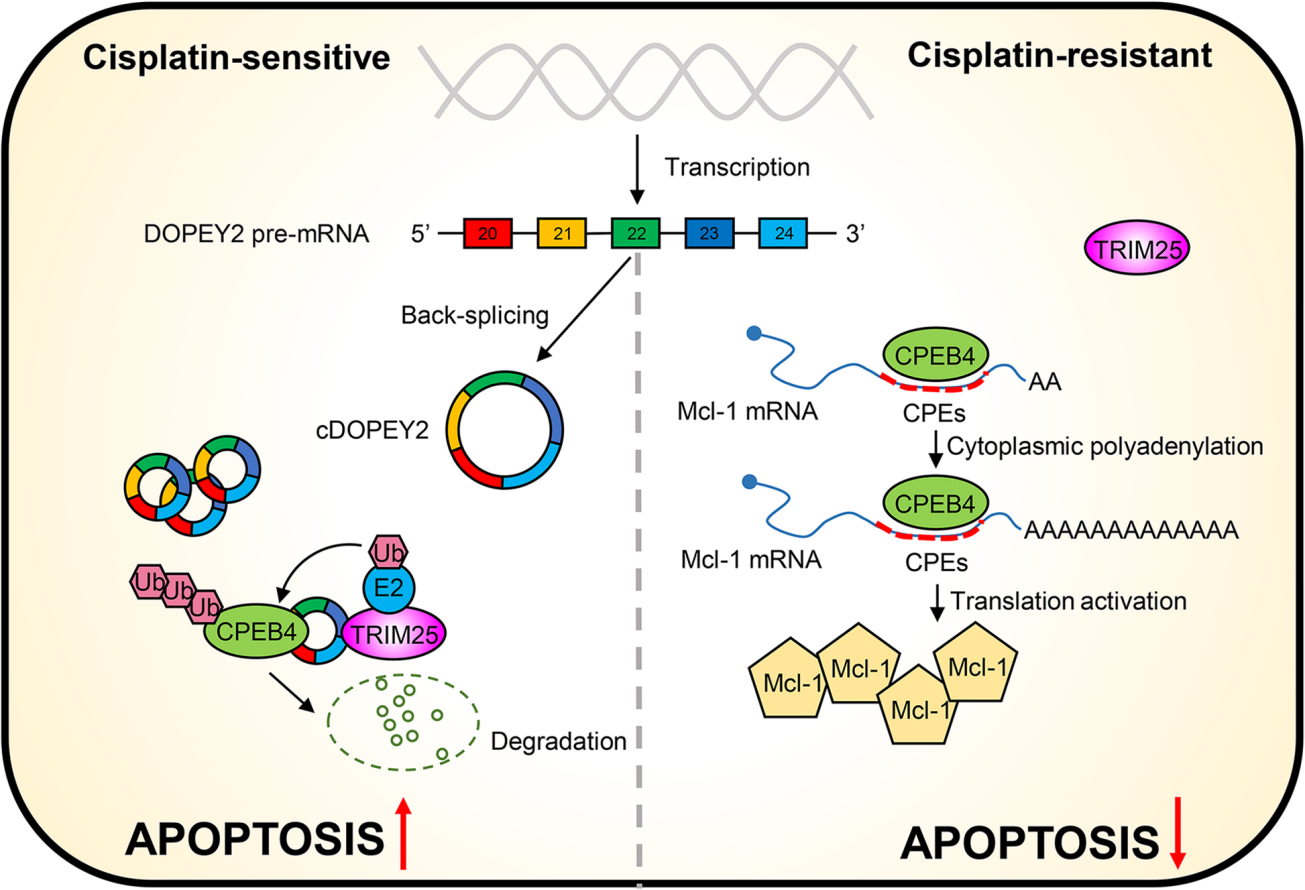
Proposed working model for the suppressive effect of cDOPEY2 on the cisplatin resistance of ESCC. cDOPEY2 originates from exons 20-24 of the DOPEY2 gene by a back-splicing event. CPEB4 promotes the translational activation of Mcl-1 by enhancing the cytoplasmic polyadenylation of its mRNA by binding to its CPEs. cDOPEY2 maintains cisplatin resistance of ESCC cells by facilitating the TRIM25-mediated ubiquitination and proteasomal degradation of CPEB4 by forming a cDOPEY2/CPEB4/TRIM25 ternary complex

Overall, our results show that cDOPEY2 acts as a scaffold to facilitate the ubiquitination and degradation of CPEB4 in a TRIM25-dependent manner, thereby boosting cisplatin-induced apoptosis by suppressing CPEB4-promoted Mcl-1 translation and alleviating cisplatin resistance in ESCC cells (Fig. 8).

## Discussion

Accumulating evidence highlights the close correlation between circRNAs and cancer development [25]. To date, although a wide range of circRNAs have been shown to be involved in the carcinogenesis, progression and metastasis of ESCC [12], chemoresistance-associated circRNAs in ESCC have rarely been reported. In the present study, we screened differentially expressed circRNAs in cisplatin-resistant ESCC cells by qPCR and analyzed microarray data. As a result, we identified a novel circRNA, cDOPEY2, that originated from the back-splicing of DOPEY2 between the 20th and 24th exons and was shown to be significantly downregulated in cisplatin-resistant cells compared with parental cells. In vivo and in vitro functional experiments demonstrated that the re-expression of cDOPEY2 made ESCC cells markedly more vulnerable to cisplatin-induced apoptosis, accompanied by the downregulation of Mcl-1. Higher cDOPEY2 levels were associated with better cisplatin responsiveness and a longer PFS for patients receiving cisplatin-based chemotherapy, as corroborated from a clinical perspective. Importantly, cDOPEY2 destabilized CPEB4 by forming cDOPEY2/CPEB4/TRIM25 RNA-protein ternary complexes, which consequently enhanced the TRIM25-mediated ubiquitination and proteasomal degradation of CPEB4. CPEB4 deficiency abrogated its ability to facilitate Mcl-1 translation, thereby leading to decreased Mcl-1 abundance and, eventually, to the sensitization of ESCC cells to cisplatin. Our data thus reveal a previously unrecognized mechanism underlying Mcl-1 regulation and provide novel evidence of the role of circRNAs in modulating cisplatin resistance.

As a newly identified type of noncoding RNA, circRNAs have been extensively investigated for their roles in cancer development. Basically, circRNAs can serve as miRNA sponges, protein scaffolds, effectors modulating the splicing and translation of mRNA, and even templates for protein translation [26]. Although most of the functions of circRNAs have been implicated in ESCC, we are the first group to report the biological relevance of circRNAs in the development and maintenance of cisplatin resistance in ESCC cells. Most studies on ESCC have focused on the role of circRNAs as miRNA sponges. Here, we demonstrated that cDOPEY2 could function not as a ceRNA but rather by destabilizing CPEB4 by acting as a protein scaffold to recruit TRIM25. CPEB4 is significantly upregulated in various types of tumors and is of paramount importance for fine-tuning the synthesis of proteins implicated in the malignancy of cancer cells [22, 27–29]. However, the regulatory mechanism of CPEB4 in tumors has rarely been explored. The present data are the first to reveal that cDOPEY2 functions as a protein scaffold to potentiate the TRIM25-induced ubiquitination of CPEB4. We also confirmed that the ubiquitin ligase activity of TRIM25 on CPEB4 relies on its RBD and on its appropriate interaction with cDOPEY2. We further elucidated that CPEB4 promotes chemoresistance by upregulating the expression of the antiapoptotic protein Mcl-1. We detected Mcl-1 overexpression in cisplatin-resistant cells at the protein rather than at the mRNA level, indicating that the alterations were not transcriptionally derived. Moreover, numerous cytoplasmic polyadenylation element motifs were observed in the 3’UTR of Mcl-1 mRNA. Consistently, our results revealed that low cDOPEY2 expression induced the activation of cytoplasmic Mcl-1 polyadenylation by CPEB4, thereby activating the translation of Mcl-1 at high levels in cisplatin-resistant cells; these phenomena caused the cells to become refractory to cisplatin-induced apoptosis and thereby promoted chemoresistance.

The antiapoptotic protein Mcl-1 is expressed in various types of malignancies, and Mcl-1 is important for the proliferation, differentiation, and survival of tumor cells because it modulates the apoptosis pathway [30–32]. Mcl-1 was shown to impair the permeabilization of the mitochondrial membrane to prevent apoptosis by sequestering BAX and BAK [15]. Importantly, high Mcl-1 expression has been observed in ESCC cell lines and primary tissues [33] and is positively correlated with cisplatin resistance [15]. Here, we further clarified that the CPEB4-promoted translation of Mcl-1 accounts for its upregulation in cisplatin-resistant cells. Our study broadens knowledge regarding the regulatory mechanism of Mcl-1 in ESCC.

Although high stability and resistance to degradation are hallmarks of circRNAs, they are generally expressed at low levels in tumors because of the potential hindrance of RNA splicing events due to an accelerated cellular proliferation rate [34], which is exactly what we observed herein. We demonstrated that cDOPEY2 expression was not only decreased in cisplatin-resistant cells but also markedly reduced in tumor tissues compared with nontumorous samples. This specific expression pattern implies that cDOPEY2 may be an ideal biomarker for identifying ESCC patients who will benefit from chemotherapy and suggests that cDOPEY2 can increase the efficacy of chemotherapy. Further exploration of the regulatory mechanism underlying the downregulation of cDOPEY2 in ESCC is clearly warranted.

## Conclusions

In conclusion, we provide the first line of comprehensive evidence that cDOPEY2 is a critical circRNA for maintaining cisplatin sensitivity as well as a diagnostic/prognostic biomarker for ESCC. cDOPEY2 plays a critical role in destabilizing CPEB4 by forming a CPEB4/cDOPEY2/TRIM25 ternary complex to promote the ubiquitination of CPEB4, which in turn antagonizes CPEB4-mediated Mcl-1 translation, thus enhancing cisplatin-induced apoptosis and chemosensitivity. Notably, our study provides a potential target, cDOPEY2, to broaden the management strategies for improving the clinical efficacy of cisplatin intervention in ESCC patients.

## Supplementary Information

**Additional file 1.**

**Additional file 2.**

## Data Availability

All data from this study can be obtained from the corresponding author upon reasonable request.

## Abbreviations

ESCC: esophageal squamous cell carcinoma
ESCC-CR: cisplatin-resistant ESCC cells
CPEB4: cytoplasmic polyadenylation element binding protein
EC: esophageal cancer
circRNAs: circular RNAs
Mcl-1: myeloid cell leukemia cell differentiation protein-1
PANTs: paired-adjacent nontumorous tissues
qPCR: quantitative polymerase chain reaction
shRNA: short hairpin RNA
DDR: DNA damage repair
PFS: progression-free survival
ORF: open reading frame
IRES: internal ribosomal entry site
MS: mass spectrum
GO: gene ontology
FISH: fluorescence in situ hybridization
RBP: RNA-binding protein
RIP: RNA immunoprecipitation
RRMs: RNA recognition motifs
CPEs: cytoplasmic polyadenylation elements
3’UTR: 3’ untranslated region
CHX: cycloheximide
IHC: immunohistochemistry

## References

[1] Thrumurthy SG, Chaudry MA, Thrumurthy SSD, Mughal M (2019). Oesophageal cancer: risks, prevention, and diagnosis. BMJ.

[2] Abnet CC, Arnold M, Wei WQ (2018). Epidemiology of Esophageal Squamous Cell Carcinoma. Gastroenterology.

[3] van Hagen P, Hulshof MC, van Lanschot JJ (2012). Preoperative chemoradiotherapy for esophageal or junctional cancer. N Engl J Med.

[4] Cao HH, Zheng CP, Wang SH (2014). A molecular prognostic model predicts esophageal squamous cell carcinoma prognosis. PLoS One.

[5] Toshimitsu H, Hashimoto K, Tangoku A (2004). Molecular signature linked to acquired resistance to cisplatin in esophageal cancer cells. Cancer Lett.

[6] Koyanagi K, Kanamori K, Ninomiya Y, et al. Progress in Multimodal Treatment for Advanced Esophageal Squamous Cell Carcinoma: Results of Multi-Institutional Trials Conducted in Japan. Cancers (Basel). 2020;13(1). 10.3390/cancers13010051 PMC7795106 33375499

[7] Maji S, Panda S, Samal SK (2018). Bcl-2 Antiapoptotic Family Proteins and Chemoresistance in Cancer. Adv Cancer Res.

[8] Galluzzi L, Senovilla L, Vitale I (2012). Molecular mechanisms of cisplatin resistance. Oncogene.

[9] Li X, Yang L, Chen LL (2018). The Biogenesis, Functions, and Challenges of Circular RNAs. Mol Cell.

[10] Du WW, Yang W, Liu E, Yang Z, Dhaliwal P, Yang BB (2016). Foxo3 circular RNA retards cell cycle progression via forming ternary complexes with p21 and CDK2. Nucleic Acids Res.

[11] Legnini I, Di Timoteo G, Rossi F (2017). Circ-ZNF609 Is a Circular RNA that Can Be Translated and Functions in Myogenesis. Mol Cell.

[12] Feng Q, Zhang H, Yao D, Chen WD, Wang YD. Emerging Role of Non-Coding RNAs in Esophageal Squamous Cell Carcinoma. Int J Mol Sci. 2019;21(1). 10.3390/ijms21010258 PMC6982002 31905958

[13] Michels J, Obrist F, Vitale I (2014). MCL-1 dependency of cisplatin-resistant cancer cells. Biochem Pharmacol.

[14] Tang Y, Yang P, Zhu Y, Su Y (2020). LncRNA TUG1 contributes to ESCC progression via regulating miR-148a-3p/MCL-1/Wnt/beta-catenin axis in vitro. Thorac Cancer.

[15] Yu X, Li W, Xia Z (2017). Targeting MCL-1 sensitizes human esophageal squamous cell carcinoma cells to cisplatin-induced apoptosis. BMC Cancer.

[16] Liu Z, Wang T, She Y (2021). N(6)-methyladenosine-modified circIGF2BP3 inhibits CD8(+) T-cell responses to facilitate tumor immune evasion by promoting the deubiquitination of PD-L1 in non-small cell lung cancer. Mol Cancer.

[17] Xu J, Ji L, Liang Y (2020). CircRNA-SORE mediates sorafenib resistance in hepatocellular carcinoma by stabilizing YBX1. Signal Transduct Target Ther.

[18] Franken NA, Rodermond HM, Stap J, Haveman J, van Bree C (2006). Clonogenic assay of cells in vitro. Nat Protoc.

[19] Gomes LR, Rocha CRR, Martins DJ (2019). ATR mediates cisplatin resistance in 3D-cultured breast cancer cells via translesion DNA synthesis modulation. Cell Death Dis.

[20] Afroz T, Skrisovska L, Belloc E, Guillen-Boixet J, Mendez R, Allain FH (2014). A fly trap mechanism provides sequence-specific RNA recognition by CPEB proteins. Genes Dev.

[21] Yang D, Liu K, Fan L (2020). LncRNA RP11-361F15.2 promotes osteosarcoma tumorigenesis by inhibiting M2-Like polarization of tumor-associated macrophages of CPEB4. Cancer Lett.

[22] Zhang Y, Gan H, Zhao F (2020). CPEB4-Promoted Paclitaxel Resistance in Ovarian Cancer In Vitro Relies on Translational Regulation of CSAG2. Front Pharmacol.

[23] Choudhury NR, Heikel G, Trubitsyna M (2017). RNA-binding activity of TRIM25 is mediated by its PRY/SPRY domain and is required for ubiquitination. BMC Biol.

[24] Pique M, Lopez JM, Foissac S, Guigo R, Mendez R (2008). A combinatorial code for CPE-mediated translational control. Cell.

[25] Geng Y, Jiang J, Wu C (2018). Function and clinical significance of circRNAs in solid tumors. J Hematol Oncol.

[26] Kristensen LS, Andersen MS, Stagsted LVW, Ebbesen KK, Hansen TB, Kjems J (2019). The biogenesis, biology and characterization of circular RNAs. Nat Rev Genet.

[27] Lu R, Zhou Z, Yu W, Xia Y, Zhi X (2017). CPEB4 promotes cell migration and invasion via upregulating Vimentin expression in breast cancer. Biochem Biophys Res Commun.

[28] Cao G, Chen D, Liu G, Pan Y, Liu Q (2018). CPEB4 promotes growth and metastasis of gastric cancer cells via ZEB1-mediated epithelial- mesenchymal transition. Onco Targets Ther.

[29] Hu J, Zhang L, Chen Q (2018). Knockdown of CPEB4 expression suppresses cell migration and invasion via Akt pathway in non-small cell lung cancer. Cell Biol Int.

[30] Lee WS, Park YL, Kim N (2015). Myeloid cell leukemia-1 regulates the cell growth and predicts prognosis in gastric cancer. Int J Oncol.

[31] Sieghart W, Losert D, Strommer S (2006). Mcl-1 overexpression in hepatocellular carcinoma: a potential target for antisense therapy. J Hepatol.

[32] Dash R, Richards JE, Su ZZ (2010). Mechanism by which Mcl-1 regulates cancer-specific apoptosis triggered by mda-7/IL-24, an IL-10-related cytokine. Cancer Res.

[33] Liu H, Yang J, Yuan Y (2014). Regulation of Mcl-1 by constitutive activation of NF-kappaB contributes to cell viability in human esophageal squamous cell carcinoma cells. BMC Cancer.

[34] Vo JN, Cieslik M, Zhang Y (2019). The Landscape of Circular RNA in Cancer. Cell.

