# Correction to: CircRNA-DOPEY2 enhances the chemosensitivity of esophageal cancer cells by inhibiting CPEB4-mediated Mcl-1 translation

**DOI:** 10.1186/s13046-022-02278-5

**Published:** 2022-02-14

**Authors:** Zhenchuan Liu, Shaorui Gu, Kaiqin Wu, Lei Li, Chenglai Dong, Wenli Wang, Yongxin Zhou

**Affiliations:** Department of Thoracic Surgery, Shanghai Tongji Hospital, School of Medicine, Tongji University, Xincun Rd. 389, 200065 Shanghai, P.R. China

**Correction to: J Exp Clin Cancer Res 40, 361 (2021)**

**https://doi.org/10.1186/s13046-021-02149-5**

Following publication of the original article [1], the authors identified a minor error in Fig. 7; specifically:

- Fig. 7d: Incorrect immunohistochemistry staining image used for CPEB4/sh-cDOPEY2 (first row, second column); correct image now used

The corrected figure is given here. The correction does not have any effect on the final conclusions of the paper. The original article has been corrected.

**Fig. 7:**
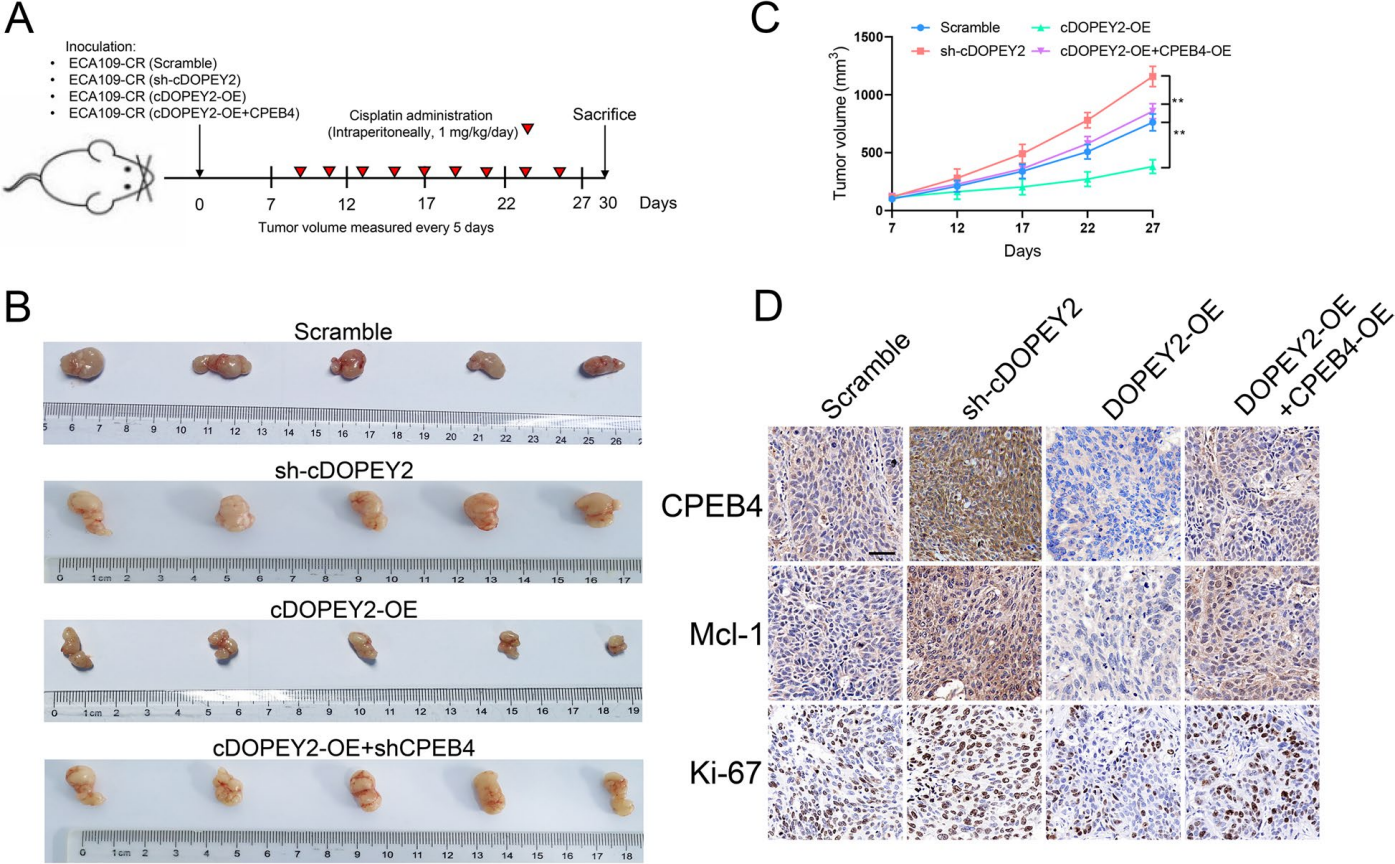
cDOPEY2 attenuates cisplatin resistance in vivo. **A** Schematic diagram demonstrating the grouping and treatment plan of the xenograft model; BALB/c nude mice were inoculated with the indicated cells (5 × 10^6^) and treated with cisplatin (i.p., 1 mg/kg) every two days until the tumor volume exceeded 100 mm^3^. **B** Representative images of the inoculated tumor tissues of each group. **C** Time-course evaluation of the tumor volumes in the indicated groups as measured every 5 days and tumor weights measured after tumors were harvested. **D** IHC staining showing the abundances of CPEB4, Mcl-1, and Ki-67 in the indicated groups. Scale bars, 100 μm. Data are presented as the mean ± SD. *P < 0.05, **P < 0.01, ***P< 0.001. P values were determined by one-way ANOVA with Tukey’s post hoc test
