# Supplementary material for: CircRNA-DOPEY2 enhances the chemosensitivity of esophageal cancer cells by inhibiting CPEB4-mediated Mcl-1 translation
Source: J Exp Clin Cancer Res. 2021 Nov 15;40:361. doi: 10.1186/s13046-021-02149-5 (PMC8591801; doi:10.1186/s13046-021-02149-5)
Supplement: Supplementary file 1 — Additional file 1. [file 13046_2021_2149_MOESM1_ESM.zip › Supplementary files.docx]

**Supplementary material for**

**CircRNA-DOPEY2 enhances the chemosensitivity of esophageal cancer cells by inhibiting CPEB4-mediated Mcl-1 translation**

**Supplementary methods and materials**

**Polysome profiling analysis**

ESCC cells were lysed in hypotonic buffer (5 mM Tris-Cl (pH 7.5), 2.5 mM MgCl2, 1.5 mM KCl, 1× protease inhibitor cocktail, 0.5% Triton X-100, 0.1 mg/mL CHX and 0.5% sodium deoxycholate) at 4°C for 20 min, followed by centrifugation at 16000×g for 7 min. Then, the lysate supernatant was collected and carefully added to the sucrose gradient solution. The samples were subjected to ultracentrifugation at 20000×g for 2 hours at 4°C, and the sucrose gradient was then collected from top to bottom. RNA was recovered from each fraction by TRIzol, and the mRNA expression of Mcl-1 in each fraction was determined by qPCR.

**Caspase-3 activity**

A Caspase-3 Activity Assay Kit (Beyotime, Shanghai, China) was used to detect caspase-3 activity according to the manufacturer's instructions. Briefly, Ac-DEVD-pNA (2 mM) was added to the cell lysis detection solution, and the absorbance at 405 nm was then measured after incubation for 2 hours at 37°C to determine caspase-3 activity.

**RNase R treatment**

Total extracted RNA was incubated for 30 min at 37°C with or without RNase R (5 U/μg RNA, Sigma-Aldrich, Munich, Germany), followed by purification using a MinElute Cleaning Kit (Qiagen, Hilden, Germany). qPCR was then performed to evaluate the stability of the RNA.

**Actinomycin D treatment**

The seeded ESCC cells were treated with 5 μg/mL actinomycin D (Sigma-Aldrich, Munich, Germany) and collected at the indicated time points. Subsequently, total RNA was extracted, and the expression of linear DOPEY2 and circ-DOPEY2 was analyzed by qPCR.

**Immunohistochemistry (IHC)**

Paraffin-embedded tissues were sectioned at a thickness of 4 μm, and the slides were then deparaffinized, rehydrated, and blocked with H_2_O_2_. Antigen retrieval was performed in TE (pH 9.0) at 100°C for 20 min. After the slides were blocked with 5% BSA, the sections were incubated with primary antibodies against CPEB4, Mcl-1, and Ki-67 at 4°C overnight followed by incubation with a biotin-conjugated secondary antibody at room temperature for 2 hours. The protein conjugates were visualized using 3,3'-diaminobenzidine (DAB) staining (Zhongshan Golden Bridge Biotechnology Company, Beijing, China), and the slides were counterstained with hematoxylin.

**Transfection**

For lentiviral-based transfection, cells seeded in 6-well plates were cultured in medium containing concentrated viral particles and polybrene (Sigma-Aldrich, Munich, Germany) for 24 hours. The infected cells were allowed to grow for another 48 hours and then selected with puromycin (1 μg/mL) (Sigma-Aldrich, Munich, Germany) for 1 week.

siRNAs targeting Mcl-1 and TRIM25 were synthesized by Sangon Biotech (Shanghai, China) and then transfected into ESCC cells using Lipofectamine 3000 (Invitrogen, MA, USA).

The oligonucleosides used in this study are listed in Table S3.

**Cycloheximide (CHX) chase assay**

Seeded cells were treated with 100 μg/mL CHX (Sigma-Aldrich, Munich, Germany) and collected at the indicated time points. Subsequently, total protein was extracted, and the expression of CPEB4 was analyzed by western blot analysis.

**Immunofluorescence (IF) and fluorescence in situ hybridization (FISH)**

For IF, the seeded cells or tissue slides were fixed with 4% paraformaldehyde, permeabilized with 0.1% Triton X-100, and blocked with 3% bovine serum albumin (BSA). Then, the cells were incubated with primary antibodies against γ-H2AX (ab81299, Abcam, Cambridge, UK), CPEB4 (ab224162, Abcam), and TRIM25 (ab167154, Abcam) at 4°C overnight followed by incubation with Alexa Fluor-conjugated secondary antibodies (ab150077 and ab150075, Abcam) at 37°C for 2 hours.

For FISH, the seeded cells were fixed, permeabilized, and prehybridized. Subsequently, Cy3-conjugated probes complementary to the back-splice site of cDOPEY2 were used to conduct hybridization. Then, the cells were rinsed with SSC and counterstained with DAPI. Images were acquired by fluorescence microscopy (Olympus, Tokyo, Japan) and analyzed with ImageJ.

**Supplementary figure legends**

**Figure S1. cDOPEY2 attenuates cisplatin resistance of ESCC cells.**

**A-B.** The relative expression of cDOPEY2 in the indicated ESCC cells transfected with plasmids expressing cDOPEY2 shRNA (**A**) and cDOPEY2 (**B**) was analyzed by qPCR. **C.** Statistical results of the clonogenic assay in the indicated ESCC cells treated with 10 μg/mL cisplatin. **D.** Statistical results of γ-H2AX staining in control or cDOPEY2-overexpressing cells treated with 10 μg/mL cisplatin. The data are presented as the mean ± SD. *P < 0.05, **P < 0.01, ***P < 0.001. P values were determined by the unpaired Student’s t-test.

**Figure S2. cDOPEY2 does not serve as a miRNA sponge or impact the expression of its host gene in ESCC.**

**A.** AGO2 RIP assay for the detection of AGO2-associated cDOPEY2 in ECA109 cells. **B.** The relative mRNA expression of linear cDOPEY2 in ESCC cells with cDOPEY2 overexpression or cDOPEY2 knockdown. The data are presented as the mean ± SD. *P < 0.05, **P < 0.01, ***P < 0.001. P values were determined by the unpaired Student’s t-test.

**Figure S3. TRIM25 promotes the degradation of the CPEB4 protein.**

**A-B.** The expression of CPEB4 in CPEB4-overexpressing or CPEB4-silencing ESCC cells was determined by qPCR (**A**) and western blotting (**B**). **C-D.** The relative expression of TRIM25 (**C**) and CPEB4 (**D**) in TRIM25-overexpressing or TRIM25-silenced ESCC cells was determined by qPCR. The data are presented as the mean ± SD. *P < 0.05, **P < 0.01, ***P < 0.001. P values were determined by the unpaired Student’s t-test.
