# Supplementary figures and images for: CircRNA-DOPEY2 enhances the chemosensitivity of esophageal cancer cells by inhibiting CPEB4-mediated Mcl-1 translation
Source: J Exp Clin Cancer Res. 2021 Nov 15;40:361. doi: 10.1186/s13046-021-02149-5 (PMC8591801; doi:10.1186/s13046-021-02149-5)

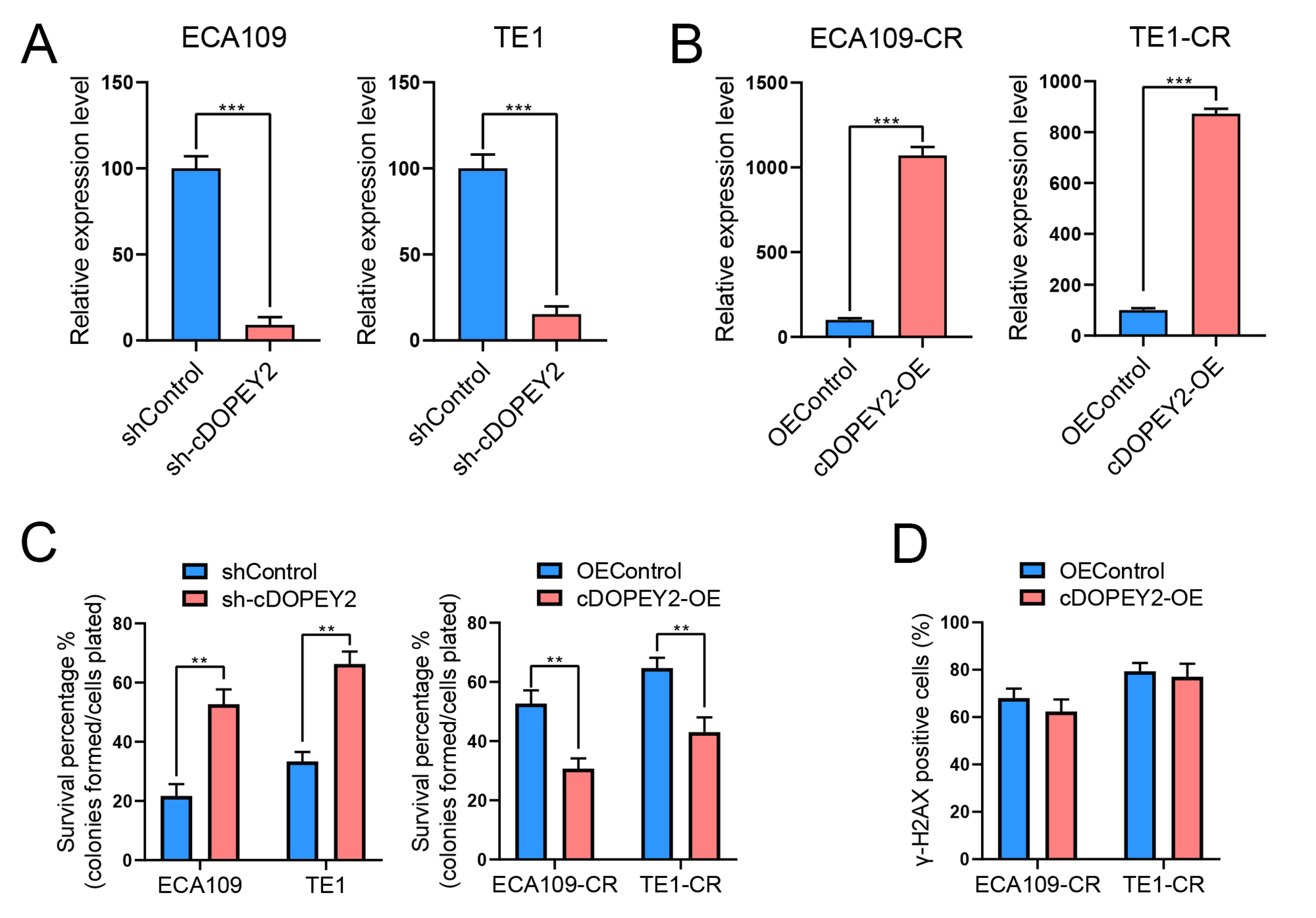

Supplement: Supplementary file 1 — Additional file 1. [file 13046_2021_2149_MOESM1_ESM.zip › FIG.S1.tif]

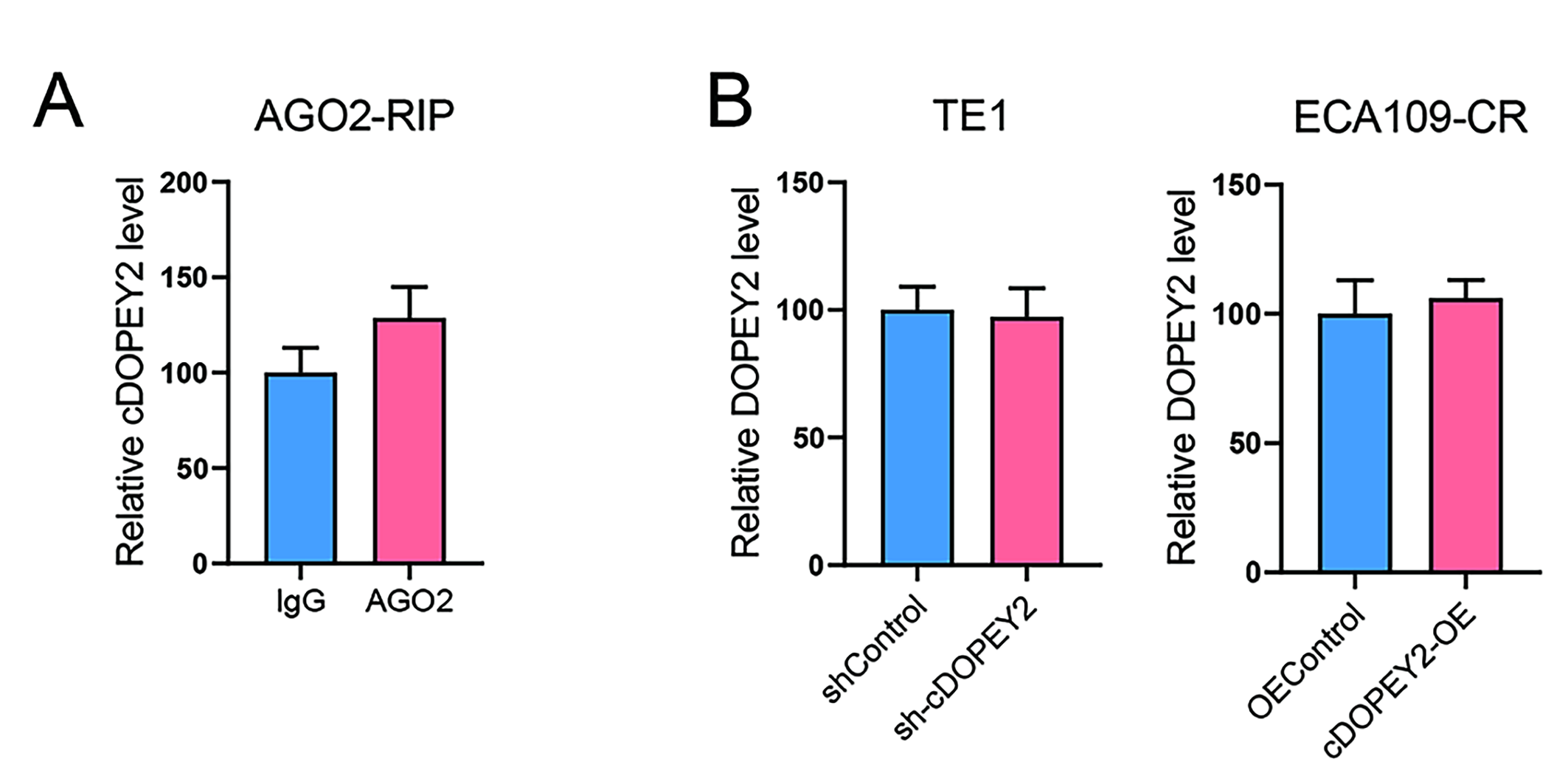

Supplement: Supplementary file 1 — Additional file 1. [file 13046_2021_2149_MOESM1_ESM.zip › FIG.S2.tif]

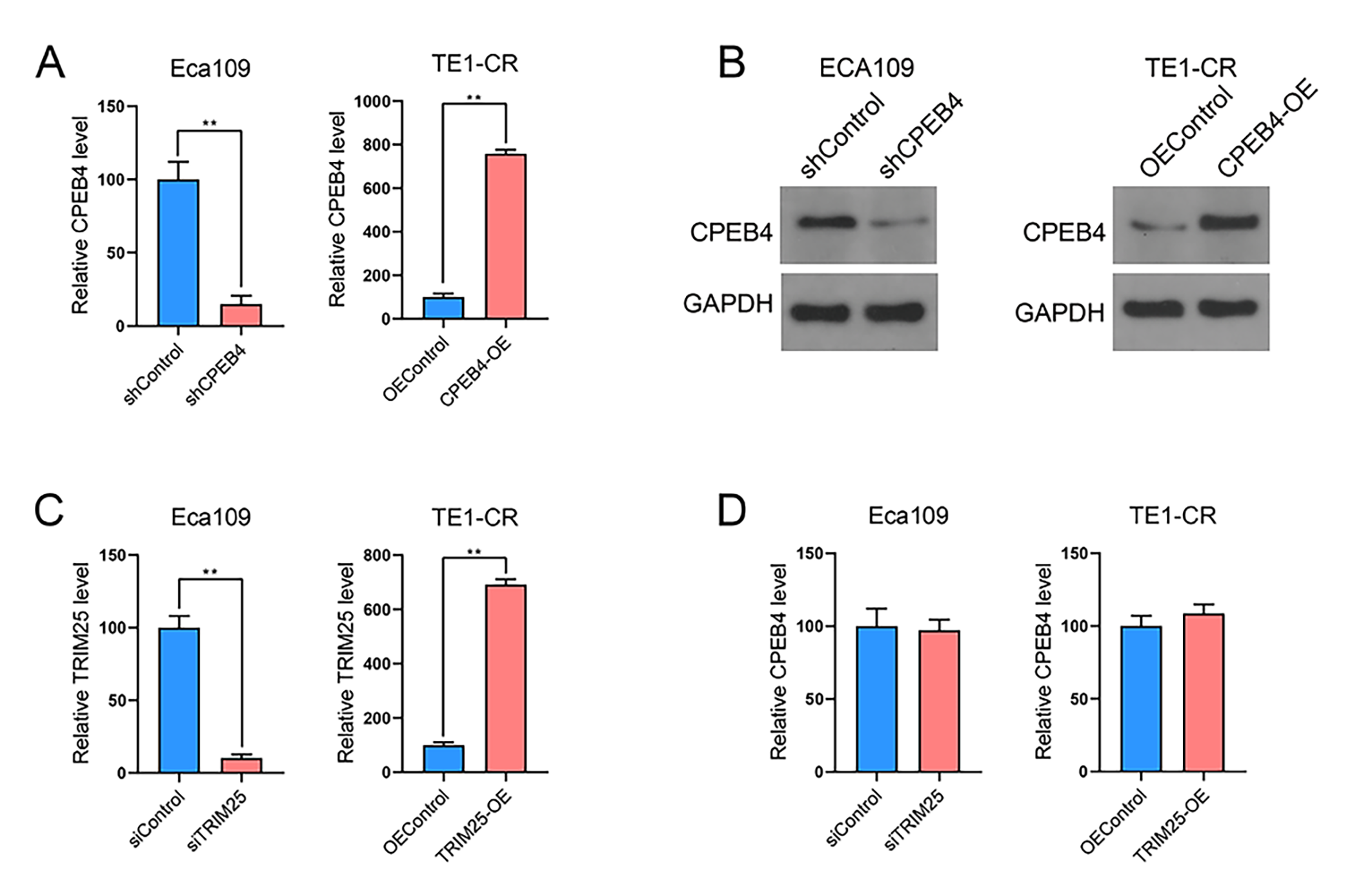

Supplement: Supplementary file 1 — Additional file 1. [file 13046_2021_2149_MOESM1_ESM.zip › FIG.S3.tif]
