# Supplementary material for: CircRNA-DOPEY2 enhances the chemosensitivity of esophageal cancer cells by inhibiting CPEB4-mediated Mcl-1 translation
Source: J Exp Clin Cancer Res. 2021 Nov 15;40:361. doi: 10.1186/s13046-021-02149-5 (PMC8591801; doi:10.1186/s13046-021-02149-5)
Supplement: Supplementary file 2 — Additional file 2. [file 13046_2021_2149_MOESM2_ESM.docx]

**Supplementary tables**

**Table S1.** Clinicopathological features of the ESCC patients in cohort I.

|  | **n = 48**  **n (%)** |
| --- | --- |
| **Age**  Median (range) | 67 (49-76) |
| **Sex**  Male  Female | 31 (64.6)  17 (35.4) |
| **Tumor location**  Upper  Middle  Lower | 13 (27.1)  24 (50.0)  11 (22.9) |
| **pT**  1  2  3  4 | 13 (27.1)  21 (43.8)  11 (15.4)  3 (6.3) |
| **pN**  0  > 0 | 38 (79.2)  10 (20.8) |
| **pM**  0  1 | 48 (100.0)  0 (0.0) |
| **TNM stage**  I  II  III  IV | 12 (25.0)  26 (54.2)  7 (14.6)  3 (6.3) |

**Table S2.** Clinicopathological features of the ESCC patients in cohort II.

|  | **n = 56**  **n (%)** |
| --- | --- |
| **Age**  Median (range) | 63 (46-79) |
| **Sex**  Male  Female | 44 (78.6)  12 (21.4) |
| **Tumor location**  Upper  Middle  Lower | 24 (42.9)  21 (37.5)  11 (19.6) |
| **pT**  3  4 | 37 (66.1)  19 (33.9) |
| **pN**  0  > 0 | 31 (55.4)  25 (44.6) |
| **pM**  0  1 | 49 (87.5)  7 (12.5) |
| **TNM stage**  III  IV | 39 (69.6)  17 (30.4) |

**Table S3.** Oligonucleotides used in this study.

| **Name** | **Sequence** | **Application** |
| --- | --- | --- |
| sh-cDOPEY2 | TTGGAGGTTGTGCTTTGGAGA | RNA interference |
| si-DOPEY2 | TCTATGAACTTCTGATGCAAT | RNA interference |
| sh-CPEB4 | CCACAGCTTCTTACTAAGTTT | RNA interference |
| si-Mcl-1 | GCTGTGTTAAACCTCAGAGTT | RNA interference |
| si-TRIM25 | GTGCCCGATTCCTCTTAGAGA | RNA interference |
| cDOPEY2 primers | F: GTCAGTGTTCACTTGCTCACAGCT | qRT-PCR |
| (Divergent) | R: AGGCTCAGTTCAAGGCTCAGGGA |  |
| cDOPEY2 primers | F: TCTGGAGAAATGTTTTCCCTCTTGG | qRT-PCR |
| (Convergent) | R: CTAGTGGACATTCCTGTGTTGCAG |  |
| hsa_circ_0007986 primers | F: CTGATTTGCTCCCTTGTTGGAGTT | qRT-PCR |
|  | R: CCCGAACTCCACCGGAGGCAATTG |  |
| hsa_circ_0055038 primers | F: CTCCATGCAAGAAAGACGCAAAGAG | qRT-PCR |
|  | R: TTATGTGATCCTTCAAACCACC |  |
| hsa_circ_0000277 primers | F: GCCAACTCCACAGCTGAGGAGAAGC | qRT-PCR |
|  | R: TATACCATTGTTTTAAGTCCCAATC |  |
| hsa_circ_0023984 primers | F: ATTGTCCCAGTGTATCTGCATTAG | qRT-PCR |
|  | R: TTGTGCTGGGTAAACTCTGCC |  |
| hsa_circ_0087378 primers | F: TATGCTGTGGTGGTGATTGCGTC | qRT-PCR |
|  | R: CTTCATAAATTACATCAGGATAAT |  |
| hsa_circ_0049613 primers | F: GTGCATCTGGAGGAACAGGACAG | qRT-PCR |
|  | R: CACCTCCAAGCTCTCAGAGCGTTC |  |
| hsa_circ_0086414 primers | F: GAAAAAGTCCTGATGAATAGTG | qRT-PCR |
|  | R: CCAGCGGTCCAGCACCTTGCCAGC |  |
| hsa_circ_0005654 primers | F: GCGTCCCTATCAATGTCCTTAC | qRT-PCR |
|  | R: TTTATTGCACACTGAACATATAAGC |  |
| hsa_circ_0000550 primers | F: TGTTCTCTCTGCTCTCTTCTGC | qRT-PCR |
|  | R: TGACGGCCACACTGCACAAGAGA |  |
| CPEB4 primers | F: AAATGATACCATTAAAGCAAGGACA | qRT-PCR |
|  | R: GGCAATCCGCCTACAAACAC |  |
| TRIM25 primers | F: CGCAAATGTTCCCAGCACAA | qRT-PCR |
|  | R: GCACCTTGGCCTTGAGAGAT |  |
| Mcl-1 primers | F: CGACTTTTGGCCACCGGC | qRT-PCR |
|  | R: GCTAGGTTGCTAGGGTGCAA |  |

**Table S4.** Antibodies used in this study.

| **Antibodies or peptides** | **Application** | **Source** | **Identifier** |
| --- | --- | --- | --- |
| Anti-γ-H2AX | WB, IF | Abcam | Cat# ab81299 |
| Anti-ATM | WB | Abcam | Cat# ab32420 |
| Anti-pATM (S1981) | WB | Abcam | Cat# ab81292 |
| Anti-CHK2 | WB | Abcam | Cat# ab109413 |
| Anti-pCHK2 (T68) | WB | Abcam | Cat# ab32148 |
| Anti-Mcl-1 | WB, IHC | Abcam | Cat# ab32087 |
| Anti-Bcl-2 | WB | Abcam | Cat#ab32124 |
| Anti-Bcl-xL | WB | Abcam | Cat#ab32370 |
| Anti-CPEB4 | WB, IF, IP | Invitrogen | Cat#PA5-115046 |
| Anti-myc-tag | WB, IP | Abcam | Cat#ab32 |
| Anti-CPEB4 | IHC | Invitrogen | Cat#PA5-25538 |
| Anti-ubiquitin | WB | Abcam | Cat#ab140601 |
| Anti-FLAG-tag | WB, IP | Abcam | Cat#ab205606 |
| Anti-TRIM25 | WB, IP, IF | Abcam | Cat#ab167154 |
| Anti-caspase-3 | WB | Abcam | Cat#ab32351 |
| Anti-cleaved caspase-3 | WB | Abcam | Cat#ab32042 |
| Anti-Ki-67 | IHC | Abcam | Cat#ab15580 |
| Anti-GAPDH | WB | Abcam | Cat#ab9485 |
